# Dietary factors and colorectal cancer risk in Asian populations: a systematic review and meta-analysis of cohort studies

**DOI:** 10.1007/s10552-025-02053-9

**Published:** 2025-08-27

**Authors:** Hyobin Lee, Linda Ngoun, Sukhong Min, Jialei Fu, Woo-Kyoung Shin, Daehee Kang, Sangah Shin

**Affiliations:** 1 Department of Preventive Medicine, Seoul National University College of Medicine, Seoul, 03080 South Korea; 2 Integrated Major in Innovative Medical Science, Seoul National University Graduate School, Seoul, 03080 South Korea; 3 Department of Food and Nutrition, Chung-Ang University, Gyeonggi-do, 17546 South Korea; 4 Vanke School of Public Health, Tsinghua University, Beijing, 100084 China; 5 Division of Food and Pharmaceutical Technology, Mokwon University, Daejeon, 35349 South Korea

**Keywords:** Food groups, Dietary pattern, Colorectal cancer, Meta-analysis, Asia

## Abstract

**Purpose:**

Colorectal cancer (CRC) is the third most common cancer worldwide and a leading cause of cancer-related mortality. Few studies have examined the preventive effects of dietary factors against CRC in Asian populations. Thus, this systematic review and meta-analysis aimed to synthesize and analyze current evidence on the relationships between various dietary components and the risk of CRC, colon cancer, and rectal cancer in Asian populations.

**Methods:**

We included cohort studies from PubMed, Web of Science, Embase, and Scopus reporting an association between dietary factors and CRC risk in Asian populations. Relative risks (RRs) with 95% confidence intervals (CIs) were used to calculate the pooled risk estimates. Cochran’s Q and I^2^ statistics were employed to assess heterogeneity. Funnel plots and Egger’s tests were used to evaluate publication bias.

**Results:**

Eighty-two publications met the inclusion criteria. The findings indicated that healthy dietary patterns were associated with a reduced risk of colon cancer (RR = 0.85, 95% CI, 0.74–0.97), and calcium intake was linked to a slight reduction in CRC risk (RR = 0.93, 95% CI, 0.86–1.00). Conversely, high total meat consumption was associated with increased risks of CRC (RR = 1.18, 95% CI, 1.03–1.34), and white meat intake was associated with a potential 40% increased risk of rectal cancer (RR = 1.40, 95% CI, 1.00–1.96).

**Conclusions:**

This review suggested that healthy dietary patterns and calcium intake are associated with a lower risk of CRC in Asian populations. Nonetheless, additional studies are warranted to clarify the associations between dietary patterns and CRC risk.

**Supplementary Information:**

The online version contains supplementary material available at 10.1007/s10552-025-02053-9.

## Introduction

Colorectal cancer (CRC) ranks as the third most common cancer and the second leading cause of cancer death worldwide [1]. The incidence of CRC has notably increased, with the number of cases worldwide having more than doubled between 1990 and 2019 [2]. Regional disparities in the CRC incidence are significant, with environmental factors presumably influencing 60 to 65% of sporadic CRC cases [3].

Lifestyle risk factors, such as smoking, sedentary behavior, obesity, alcohol consumption, and dietary habits, have been well-documented [4]. The dietary association with various cancers, especially CRC, is well-established. On October 26, 2015, the International Agency for Research on Cancer (IARC) designated processed meat as carcinogenic (Group 1) to humans and unprocessed red meat as likely carcinogenic (Group 2A) [5]. Moreover, the 2018 report by the World Cancer Research Fund/American Institute of Cancer Research (WCRF/AICR) Panels concluded that consuming processed meat increases the risk of CRC, similar to red meat consumption [6]. They also concluded that foods containing whole grains, dietary fiber, dairy products, and calcium supplements are protective against CRC [6]. However, these findings are predominantly based on studies from historically high-incidence Western countries, with 76% of the studies referenced by the IARC conducted in Western nations, and a mere 15% in Asia [7].

In Asia, where CRC has become more pronounced, fewer studies have been conducted on the impact of diet on CRC among these populations [8]. Regional data indicate that Asia bears a significant burden of new CRC cases, deaths, and 5-year prevalence rates [9]. Notably, East, South, and Southeast Asia have experienced a 2- to fourfold increase in the crude incidence rates of CRC [10]. The “westernization” of dietary patterns in many Asian countries is an ongoing trend, yet the distinct nature of food definitions, types, preparations, and cooking methods in these regions could contribute to divergent results [8, 11]. Notably, the annual consumption of processed meat products per capita was calculated to be one-seventh that of Germany [7]. The physiological responses to dietary components may also vary in the Asian population, underscoring the need for targeted evaluations [12].

To date, our knowledge is limited to one systematic review [8] and one scoping review [13] that examined the epidemiological links between all food groups and CRC risk in Asian populations, with only one meta-analysis focused exclusively on Chinese populations [14]. Therefore, this systematic review and meta-analysis aimed to synthesize and analyze current evidence on the relationships between various dietary components and the risk of CRC, colon cancer, and rectal cancer in Asian populations.

## Materials and methods

### Search strategy

The Preferred Reporting Items for Systematic Reviews and Meta-Analyses (PRISMA) guidelines [15] were applied in this systematic review and meta-analysis (Appendix 1). Relevant studies restricted to English and published up to November 2023 were searched in the PubMed, Web of Science, Embase, and Scopus databases with the search strategy provided in Supplementary Table S1.

### Eligibility criteria

Publications were selected based on the following inclusion criteria (Supplementary Table S2): 1. Studies focused on healthy adults among Asian populations, with no restrictions on study population sizes. 2. The incidence of CRC, colon cancer, and rectal cancer was considered the outcome. For example, cancer recurrence or mortality as an outcome was not considered as the outcome. 3. Only cohort studies were considered and included in the study. Thus, the following exclusion criteria were applied: 1. Studies conducted on or involving nonhuman participants. 2. Systematic review, meta-analysis, narrative review, or other review-type studies. 3. Other types of cancer incidence as outcomes. Two authors (H.L. and L.N.) screened the titles and abstracts of the studies fulfilling the inclusion criteria, followed by careful screening of the full texts, with disagreements or inconsistencies resolved by the consensus of a third author (S.S.).

### Data extraction and quality assessment

Two authors (H.L. and L.N.) extracted the following information from each including publication: first author, year of publication, country, duration of follow-up, exposure intake of study, outcome, outcome assessment, type and specification of food group, and potential adjustment factors. The quality of the included studies was evaluated using the Newcastle–Ottawa Scale criteria for cohort studies (Appendix 2). Two authors (H.L. and L.N.) independently evaluated the following three domains: selection, comparability, and outcome. In addition, if the quality of the evidence (out of 9 points) was more than 7 points, the study was allocated as high-quality evidence.

### Statistical analysis

Hazard ratios (HRs) or relative risks (RRs) with 95% confidence intervals (CIs) were extracted and then pooled for data analysis by random-effects models. For consistency in reporting, all effect estimates were expressed as RRs. Despite their asymmetry, log-transformed (base 10) values were calculated [16]. For quantitative analysis of heterogeneity, studies were assessed using Cochran’s Q test and the I^2^ statistic with an I^2^ value > 50% or a *p*-value < 0.05, indicating significant heterogeneity. Additionally, subgroup analyses were conducted to explore the stability of the primary results. Moreover, when the number of included studies was ≥ 10, a funnel plot was used to detect publication bias. In this review, funnel plot asymmetry was assessed by the Egger’s test and Begg’s test for publication bias [17]. All the statistical analyses were computed using STATA SE 17.0, with a two-tailed *p*-value < 0.05 considered significant.

## Results

### Study characteristics and quality assessment

**Fig. 1:**
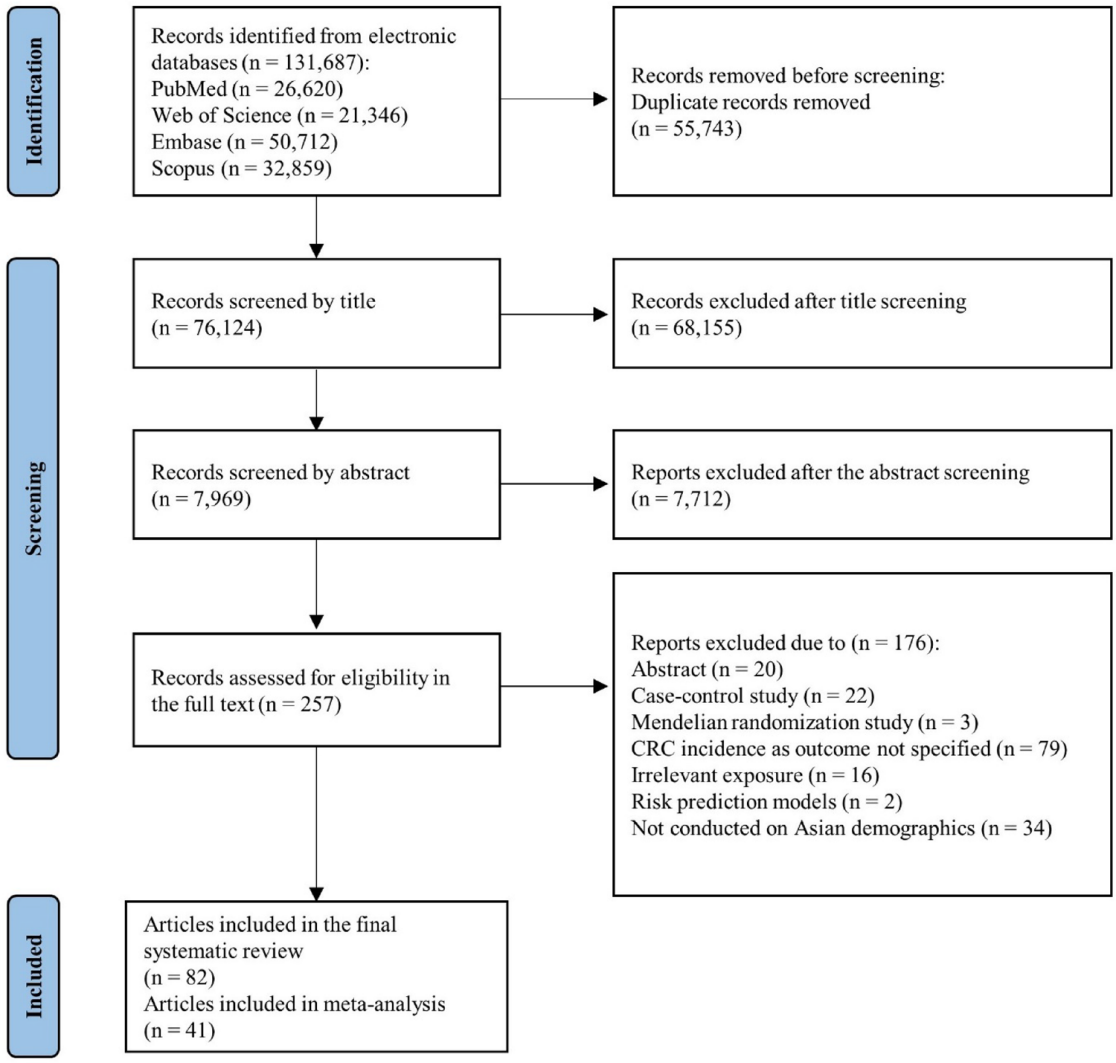
Flow diagram of selection criteria

An extensive primary search across four distinct databases generated 131,687 entries (Fig. 1). After pooling records from the four databases, 55,743 duplicates were excluded. A total of 76,124 publications were first examined based on their titles, and an additional 7,969 publications were evaluated based on their abstracts. Following both screenings, 258 publications were evaluated for eligibility based on the full text. The final systematic review included 82 publications after comprehensively evaluating the full texts. The publications examined the associations between the risk of CRC and various exposures, including alcohol (*n* = 14), coffee or tea (*n* = 13), meat (*n* = 5), dairy (*n* = 1), fish or fatty acids (*n* = 7), fruits or vegetables (*n* = 7), soy food (*n* = 4), carbohydrates (*n* = 8), micronutrients (*n* = 8), dietary fiber (*n* = 3), dietary patterns (*n* = 5), spicy food (*n* = 1), ginseng (*n* = 1), and multiple food groups (*n* = 5) consumptions. The characteristics and quality scores of the publications included are described in Supplementary Tables S3 and S4, respectively. Also, a summary of included cohort studies and dietary and drinking habit assessment methods is described in Supplementary Table S5.

In this review, we defined a publication as a peer-reviewed article and a study as a distinct analytical unit extracted from each publication. A single publication could contribute multiple studies if it reported separate risk estimates by cancer site (colorectal, colon, or rectal), or sex (men or women). For example, if one publication reported RRs separately for men and women across three cancer outcomes, it was treated as six separate studies.

Meta-analyses were primarily conducted when the total number of available studies combining colorectal, colon, and rectal cancer outcomes were five or more. However, for some subgroup analysis, particularly those focused on site-specific outcomes such as colon or rectal cancer, fewer studies were available. These subgroup results were still presented to provide a more comprehensive summary of the evidence and to support comparisons across different cancer subtypes. Moreover, if multiple publications included a specific cohort study (e.g., the Japan Public Health Cohort (JPHC) study) for a given exposure factor, only the RR values provided in the most recent publication were used for the meta-analysis. Additionally, for each exposure factor, publications were selected to include results from the largest possible number of cohorts. For example, in the case of alcohol, the meta-analysis included the RR values from a publication which pooled the JPHC, Japan Collective Cohort (JACC), Miyagi, and Takayama cohorts, as well as RR values extracted from individual publications from the Korean Multicenter Cancer Cohort (KMCC), National Health Insurance System (NHIS) database, and China Kadoorie Biobank (CKB) databases. Among the 82 publications, the RRs from 41 publications were subsequently used for meta-analysis. The 41 publications used in the meta-analysis investigated the associations between colorectal, colon and rectal cancers and consumption of total meat (*n* = 5), red meat (*n* = 5), processed meat (*n* = 3), white meat (*n* = 2), fruits and vegetables (*n* = 3), fruits (*n* = 5), vegetables (*n* = 5), soy food (*n* = 4), soy isoflavone (*n* = 2), fish (*n* = 4), alcohol (*n* = 5), green tea (*n* = 7), coffee (*n* = 4), calcium (*n* = 4), fiber (*n* = 4), and dietary patterns (*n* = 3). Since some publications examined multiple dietary factors, the sum of these numbers exceeds the total number of 41 publications.

### Meta-analyses

#### Meat

##### Total meat

**Fig. 2:**
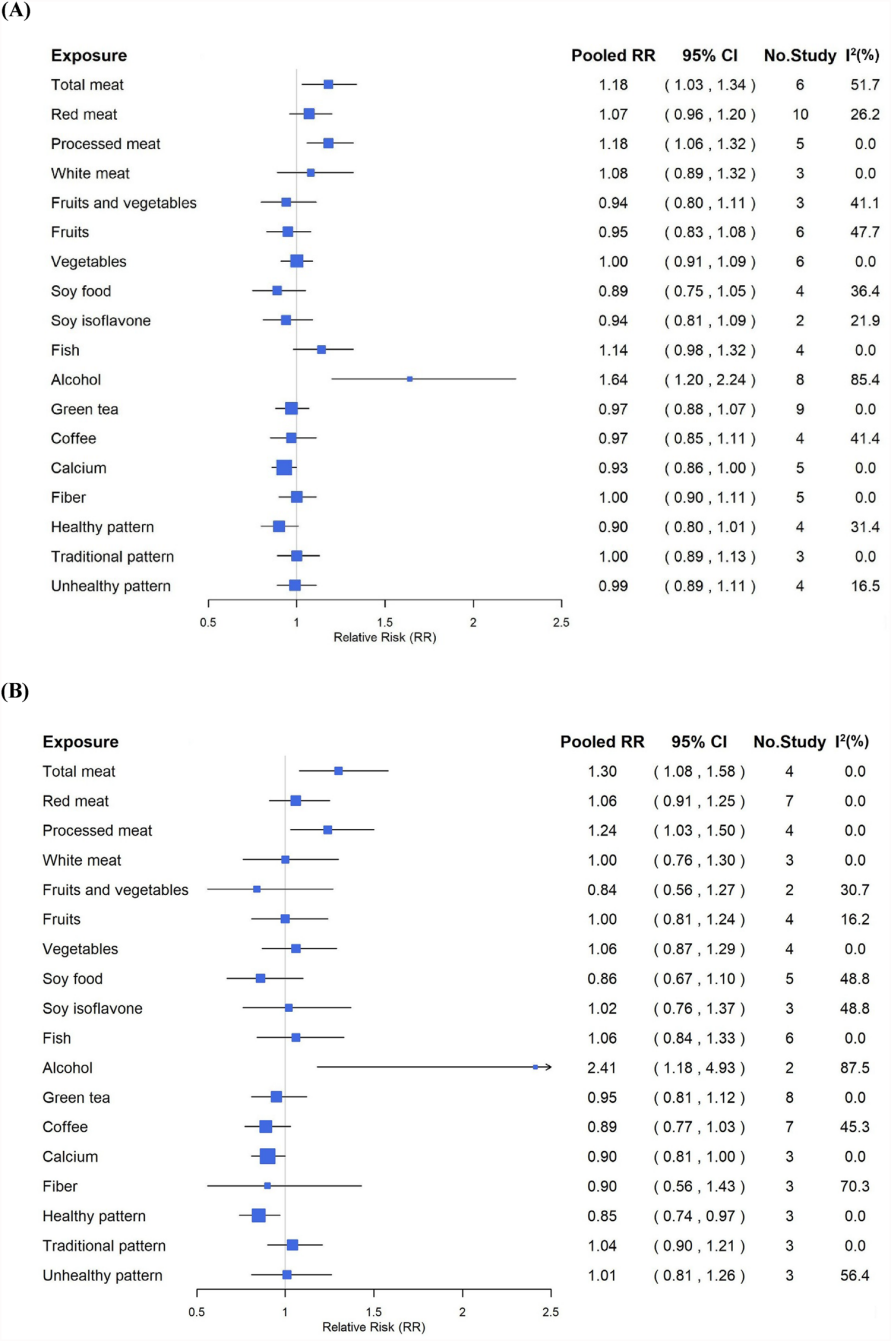

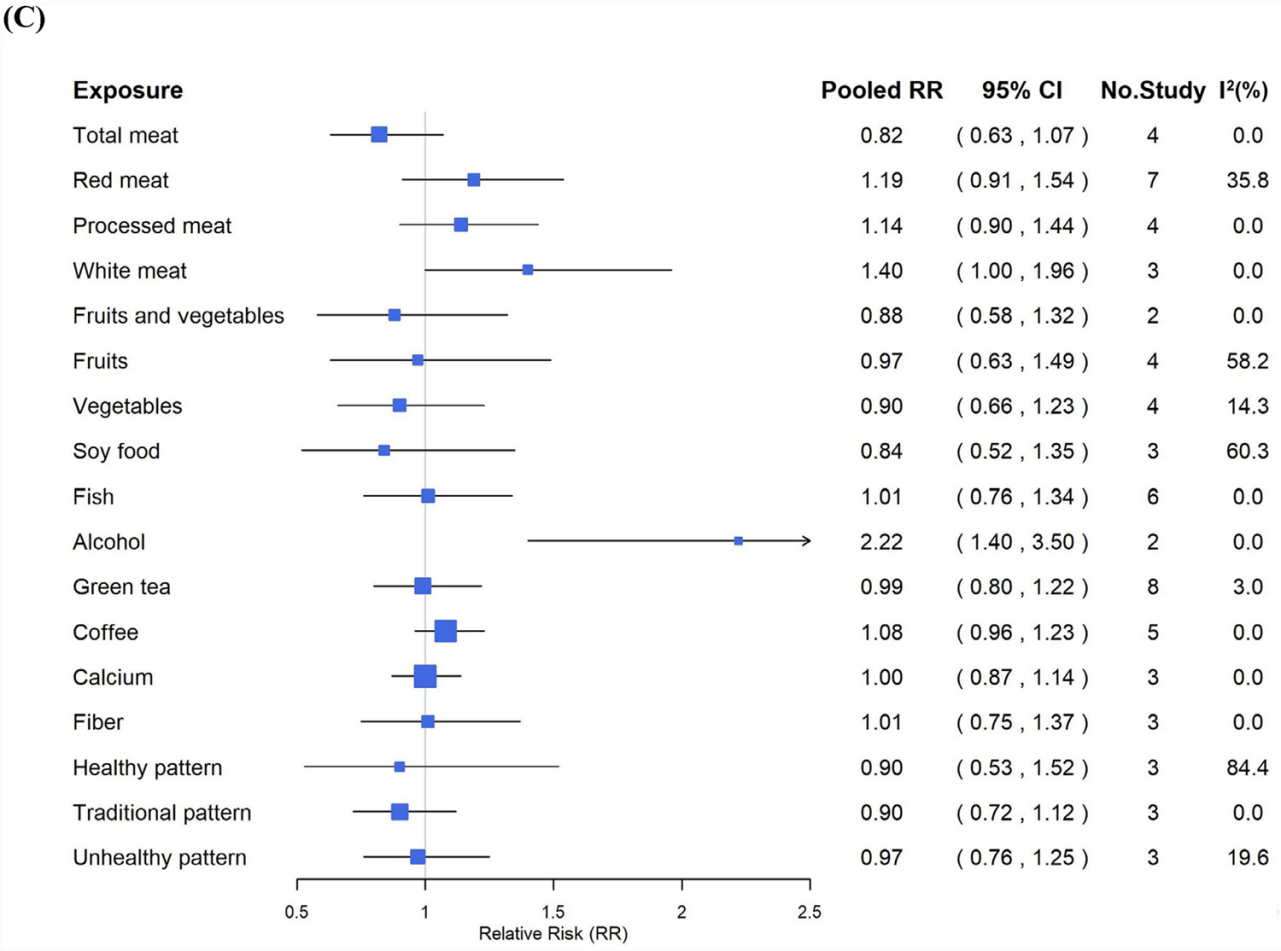
Pooled results of food groups, dietary patterns, and **A** colorectal cancer, **B** colon cancer, and **C** rectal cancer risk

Five publications were included in the meta-analysis [18–22]. Total meat intake in the highest categories ranged from ≥ 89 to 114 g/day across included publications. Extracted from the publications, six studies showed that high consumption of total meat was associated with CRC risk with a pooled RR = 1.18 (95% CI, 1.03–1.34) and high heterogeneity (I^2^ = 51.7%; *p* = 0.066) (Fig. 2, Supplementary Figure S1). Four studies revealed that the pooled RR of colon cancer was also significant (RR = 1.30, 95% CI, 1.08–1.58), with no heterogeneity (I^2^ = 0.0%); contrarily, four studies revealed that total meat consumption was not associated with rectal cancer risk (RR = 0.82, 95% CI, 0.63–1.07) without heterogeneity.

##### Red meat

Five publications were included in the analysis[19, 21, 23–25]. Red meat intake among the highest categories ranged from 67 to ≥ 74 g/day. Extracted from the publications, ten studies that examined the high consumption of red meat were associated with CRC risk with a pooled RR = 1.07 (95% CI, 0.96–1.20), with low heterogeneity (I^2^ = 26.2%; *p* = 0.202) (Fig. 2, Supplementary Figure S1). The pooled results of seven studies also demonstrated no association with colon cancer risk (RR = 1.06, 95% CI, 0.91–1.25), with no heterogeneity, whereas seven studies revealed the pooled RR of rectal cancer with red meat consumption was 1.19 (95% CI, 0.91–1.54), with low heterogeneity (I^2^ = 35.8%; *p* = 0.155).

##### Processed meat

Three publications were included in the analysis [21, 23, 25]. Processed meat intake in the highest categories ranged from 8.5 to 23 g/day. Extracted from the publications, five studies revealed that high consumption of processed meat was a significant risk factor for CRC (RR = 1.18, 95% CI, 1.06–1.32), with no heterogeneity observed (Fig. 2, Supplementary Figure S1). The pooled result of colon cancer from four studies also estimated significant risk (RR = 1.24, 95% CI, 1.03–1.50) with no heterogeneity. Meanwhile, the pooled results of four studies of processed meat consumption demonstrated no association with rectal cancer (RR = 1.14, 95% CI, 0.90–1.44) with no heterogeneity.

##### White meat

Two publications were included in the analysis [19, 23]. White meat consumption in the highest category in a publication was 24 g/day or more, while another publication reported almost every day as the highest category. In the included studies, white meat was defined as poultry, such as chicken or turkey, and did not include fish. Extracted from the publications, three studies revealed that white meat consumption was not significantly associated with CRC or colon cancer risk (CRC: RR = 1.08, 95% CI, 0.89–1.32; colon cancer: RR = 1.00, 95% CI, 0.76–1.30), with no heterogeneity (Fig. 2, Supplementary Figure S1). In contrast, three studies reported that the pooled RR of white meat with rectal cancer was 1.40 (95% CI, 1.00–1.96), without heterogeneity (I^2^ = 0.0%).

#### Fruits and vegetables

Three publications were included in the analysis [26–28]. For fruits and vegetables, the intake in the highest categories ranged from 495.2 to 698 g/day. Extracted from the publications, three studies evaluated the associations between the consumption of total fruits and vegetables and CRC risk, which was not significant (RR = 0.94, 95% CI, 0.80–1.11), with heterogeneity (I^2^ = 41.1%; *p* = 0.183) (Fig. 2, Supplementary Figure S1). Similarly, the pooled results from two studies for total fruits and vegetables with colon cancer were not significant (RR = 0.84, 95% CI, 0.56–1.27), with low heterogeneity (I^2^ = 30.7%; *p* = 0.23), and the pooled result of two studies for total fruits and vegetables with rectal cancer was also not significant (RR = 0.88, 95% CI, 0.58–1.32), with no heterogeneity observed (I^2^ = 0.0%).

##### Fruits

Five publications were included in the analysis[25–29]. Fruit intake in the highest categories across these publications ranged from 242 to 472 g/day. Extracted from the publications, six studies evaluated the associations between the consumption of fruits and CRC risk. High consumption of fruits was not associated with CRC risk, with a pooled RR 0.95 (95% CI, 0.83–1.08), with moderate heterogeneity observed (I^2^ = 47.7%; *p* = 0.089) (Fig. 2, Supplementary Figure S1). Thus, four studies demonstrated no association between fruits consumption and colon cancer risk (RR = 1.00, 95% CI, 0.81–1.24) with low heterogeneity (I^2^ = 16.2%; *p* = 0.31). Furthermore, the pooled result of four studies of rectal cancer risk was not significantly associated with fruit consumption (RR = 0.97, 95% CI, 0.63–1.49), with high heterogeneity (I^2^ = 58.2%; *p* = 0.066).

##### Vegetables

Five publications were included in the analysis[25–29]. Among the highest intake categories, vegetable consumption ranged from 208 to 466.6 g/day across publications. Extracted from the publications, the pooled results of six studies examined that the high consumption of vegetables was not associated with CRC risk (RR = 1.00, 95% CI, 0.91–1.09), with no heterogeneity observed (Fig. 2, Supplementary Figure S1). Similarly, the pooled result of four studies also revealed no association with colon cancer risk (RR = 1.06, 95% CI, 0.87–1.29), with no heterogeneity; however, the pooled RR of four studies of vegetable consumption with rectal cancer was 0.90 (95% CI, 0.66–1.23), with low heterogeneity (I^2^ = 14.3%; *p* = 0.321).

#### Soy foods and soy isoflavone

Four publications were included in the analysis [25, 30–32]. Across the highest intake categories, soy food consumption was reported between 170 and 254 g/day. Extracted from the publications, four studies examined the association between soy foods consumption and CRC risk. The consumption of soy foods was not significantly associated with CRC risk (RR = 0.89, 95% CI, 0.75–1.05), and low heterogeneity was detected (I^2^ = 36.4%; *p* = 0.194) (Fig. 2, Supplementary Figure S1). This non-significant association was also observed from the pooled RR from five studies, between soy foods and colon cancer (RR = 0.86, 95% CI, 0.67–1.10), with moderate heterogeneity observed (I^2^ = 48.8%; *p* = 0.099). Also, the pooled RR from three studies that examined the association between soy foods and rectal cancer (RR = 0.84, 95% CI, 0.52–1.30) was non-significant with high heterogeneity (I^2^ = 60.3%; *p* = 0.08).

Also, extracted from two publications [30, 33], the pooled RR of two studies that investigated soy isoflavone consumption for CRC risk was 0.94 (95% CI, 0.81–1.09), with low heterogeneity (I^2^ = 21.9%; *p* = 0.258), and the pooled RR of three studies for colon cancer was 1.02 (95% CI, 0.76–1.37, I^2^ = 48.8%; *p* = 0.142). Soy isoflavone intake of the highest category ranged from 59.5 to > 60.9 mg/day.

#### Fish

Four publications were included in the analysis[25, 34–36]. In the highest intake categories, fish consumption ranged from more than 122.5 to 195.7 g/day. Extracted from the publications, the pooled results of four studies demonstrated that high consumption of fish was not significantly associated with CRC risk (RR = 1.14, 95% CI, 0.98–1.32), with no heterogeneity (Fig. 2, Supplementary Figure S1). Moreover, the pooled results of six studies revealed no association with colon cancer risk (RR = 1.06, 95% CI, 0.84–1.33), with no heterogeneity, while the pooled RR from six studies revealed no association with of rectal cancer risk (95% CI, 0.76–1.34), without heterogeneity.

#### Alcohol

Five publications were included in the analysis[37–41]. Among the highest intake categories, alcohol consumption ranged from more than 30 g/day to more than 92 g/day. Extracted from the publications, eight studies assessed the associations between alcohol consumption and CRC. The pooled RR for CRC was 1.64 (95% CI, 1.20–2.24), with high heterogeneity (I^2^ = 85.4%; *p* = 0.00%) (Fig. 2, Supplementary Figure S1). Similarly, the pooled RR for colon cancer, revealed from two studies was 2.41 (95% CI, 1.18–4.93), demonstrating high heterogeneity (I^2^ = 87.5%; *p* = 0.005). The pooled RR from two studies, for the association with alcohol consumption and rectal cancer was 2.22 (95% CI, 1.40–3.50), with no heterogeneity.

#### Green tea

Seven publications were included in the analysis [42–48]. In the highest intake categories, green tea consumption ranged from 3 cups/day to 5 or more cups/day. Extracted from the publications, the pooled result of nine studies that observed the association of green tea with CRC examined no significance (RR = 0.97, 95% CI, 0.88–1.07), with no heterogeneity (Fig. 2, Supplementary Figure S1). Similarly, the pooled RR of eight studies for colon cancer was 0.95 (95% CI, 0.81–1.12), with no heterogeneity, while the pooled RR of eight studies for rectal cancer was 0.99 (95% CI, 0.80–1.22), with low heterogeneity observed (I^2^ = 3.0%; *p* = 0.407).

#### Coffee

Four publications were included in the analysis[48–51]. In the highest categories across publications, coffee intake ranged from 2 cups/day to 3 or more cups/day. Extracted from the publications, the summary pooled result of four studies that observed the association of coffee with CRC risk indicated no statistical significance (RR = 0.97, 95% CI, 0.85–1.11), with low heterogeneity (I^2^ = 41.4%; *p* = 0.163) (Fig. 2, Supplementary Figure S1). In addition, pooled RR of seven studies revealed no association between coffee and colon cancer (RR = 0.89, 95% CI, 0.77–1.03), with low heterogeneity (I^2^ = 45.3%; *p* = 0.089), while the pooled result of five studies between coffee and rectal cancer was 1.08 (95% CI, 0.96–1.23), with no heterogeneity.

#### Calcium

Four publications were included in the analysis[25, 52–54]. The publications assessed calcium from dietary sources only, and none incorporated supplemental calcium in quantitative analysis. In the highest categories, dietary calcium intake ranged from 661 to 800 mg/day. Extracted from the publications, five studies examined the association between calcium intake and CRC with a pooled RR of 0.93 (95% CI, 0.86–1.00), with no heterogeneity (Fig. 2, Supplementary Figure S1). This result suggests that calcium consumption may potentially lower CRC risk, although the result was not statistically significant. The pooled results of three studies that observed the association of calcium intake and colon cancer also obtained a similar RR of 0.90 (95% CI, 0.81–1.00), without heterogeneity. Moreover, the pooled results of three studies found no significant association between calcium intake and rectal cancer (RR = 1.00, 95% CI, 0.87–1.14), with no heterogeneity observed (I^2^ = 0.0%).

#### Fiber

Four publications were included in the analysis [28, 52, 55, 56]. Fiber intake in the highest categories ranged from 18.7 to 20 g/day across the included publications. Extracted from four publications, none of the pooled RR estimates for fiber consumption were significantly associated with CRC, colon cancer, or rectal cancer (Fig. 2, Supplementary Figure S1). There was no association between the CRC risk and fiber consumption, revealed from the pooled results of five studies (RR = 1.00, 95% CI, 0.90–1.11, I^2^ = 0.0%). The pooled RR result of three studies for colon cancer demonstrated no reduction in the risk with substantial heterogeneity (RR = 0.90, 95% CI, 0.56–1.43, I^2^ = 70.3%; *p* = 0.034). Also, three studies also revealed an insignificant association between fiber and rectal cancer, a with pooled RR of 1.01 (95% CI, 0.75–1.37), without heterogeneity.

#### Dietary pattern

Considering the loading factors of each dietary pattern and food group, vegetable-based dietary patterns were unified as the “healthy pattern,” whereas animal food-based dietary patterns were unified as the “unhealthy pattern.” Moreover, the traditional Asian dietary pattern characterized by high intakes of fish, seaweeds, salted vegetables, and fruits was unified as the “Traditional pattern.” A total of three publications was include in the meta-analysis [25, 57, 58].

##### Healthy pattern

The pooled results based on four studies showed that the healthy pattern was associated with a non-significant reduction in the risk for CRC with a pooled RR of 0.90 (95% CI, 0.80–1.01) and low heterogeneity (I^2^ = 31.4%; *p* = 0.224) (Fig. 2, Supplementary Figure S1). In addition, the pooled RR of three studies of the healthy pattern for colon cancer was 0.85 (95% CI, 0.74–0.97), with no observed heterogeneity (I^2^ = 0.0%), indicating a significant 15% reduction in risk. Three studies reported that the overall association between healthy patterns and rectal cancer was 0.90 (95% CI, 0.53–1.52), with high heterogeneity (I^2^ = 84.4%; *p* = 0.002).

##### Traditional pattern

Three studies evaluated the impact of a traditional pattern on CRC risk. The pooled RR was 1.00 (95% CI, 0.89–1.13), indicating that the traditional pattern did not affect the CRC risk. Heterogeneity among these studies was not observed (Fig. 2, Supplementary Figure S1). However, three studies investigated the association between traditional patterns and CRC risk, with a pooled RR of 1.04 (95% CI, 0.90–1.21), with no heterogeneity or significant associations. Three studies investigated the association of traditional patterns with rectal cancer, resulting in a pooled RR of 0.90 (95% CI, 0.72–1.12), indicating no significant association. This indicates no significant association between a traditional pattern and rectal cancer risk. There was no heterogeneity among the studies (I^2^ = 0.0%).

##### Unhealthy pattern

Four studies investigated the relationship between unhealthy dietary patterns and CRC risk. The pooled RR was found to be 0.99 (95% CI, 0.89–1.11), with low heterogeneity (I^2^ = 16.5%; *p* = 0.309) (Fig. 2, Supplementary Figure S1). This suggests that there is no significant increase or decrease in the risk of CRC associated with an unhealthy pattern. For colon cancer, the pooled RR of three studies was 1.01 (95% CI, 0.81–1.26), indicating no significant association between an unhealthy pattern and colon cancer risk, with slightly high heterogeneity (I^2^ = 56.4%; *p*= 0.101). In the case of rectal cancer, three studies were analyzed, resulting in a pooled RR of 0.97 (95% CI, 0.76–1.25). Additionally, the analysis demonstrated moderate heterogeneity (I^2^ = 19.6%; *p* = 0.23), suggesting no significant association between an unhealthy pattern and rectal cancer risk.

### Subgroup analysis

The combined findings of alcohol, coffee, total meat, fruits, soy food, fiber, and healthy patterns demonstrated significant heterogeneity (Fig. 2, Supplementary Table S6). Subgroup analyses were conducted based on sex, country, and number of participants (Table 1). Studies among male participants revealed a significant risk association between total meat consumption and CRC, resulting in a pooled RR of 1.18 (95% CI, 1.01–1.39, I^2^ = 33.6%) and 0.85 (95% CI, 0.74–0.97, I^2^ = 0.0%) for colon cancer. Regarding total meat consumption, studies in Japan and Korea revealed a greater risk between total meat consumption and CRC, with a pooled RR of 1.35 (95% CI, 1.09–1.66, I^2^ = 0.0%) and 1.26 (95% CI, 1.01–1.57, I^2^ = 82%) for colon cancer, respectively. Moreover, studies with sample sizes of greater than 50,000 participants also suggested the possibility of increased risk of colon cancer (RR = 1.33, 95% CI, 1.08–1.65) with no heterogeneity. One study in China demonstrated a significant association between soy food consumption and CRC and rectal cancer risk, with RRs of 0.67 (95% CI, 0.49–0.91) and 0.55 (95% CI, 0.34–0.89), respectively. A study with a sample size of fewer than 50,000 participants showed that fiber consumption was associated with a significantly reduced risk for colon cancer (RR = 0.58, CI = 0.38–0.88). Research on coffee consumption in Japan demonstrated a statistically significant risk reduction, with a pooled RR of 0.91 (95% CI, 0.83–1.00, I^2^ = 0.0%) for CRC and 0.82 (95% CI, 0.67–0.99, I^2^ = 41.8%) for colon cancer. Studies conducted in Japan revealed a noteworthy correlation between healthy patterns and a lower risk for CRC (pooled RR = 0.86 [95% CI, 0.76–0.98], I^2^ = 15.2%) and colon cancer (pooled RR = 0.85 [95% CI, 0.74–0.97], I^2^ = 0.0%). Thus, studies with sample sizes greater than 50,000 participants also suggested the possibility of lowering colon cancer risk by 17% (RR = 0.83, 95% CI, 0.72–0.97), with no heterogeneity. In contrast, studies with sample sizes of fewer than 50,000 participants each demonstrated a reduction in the risk of CRC and rectal cancer, with pooled RRs of 0.76 (95% CI, 0.60–0.97) and 0.56 (95% CI, 0.37–0.84), respectively.

**Table 1:** Stratified analyses of food groups, dietary patterns and colorectal cancer risk

	Subgroup	Colorectal cancer			Colon cancer			Rectal cancer		
		No. Study	RR (95% CI)	I^2^	No. Study	RR (95% CI)	I^2^	No. Study	RR (95% CI)	I^2^
*Total meat*										
Sex	Men	2	1.18 (1.01, 1.39)	33.6	1	1.44 (1.05, 1.97)	–	1	0.83 (0.52, 1.31)	–
	Women	3	1.12 (0.83, 1.52)	73.9	2	1.24 (0.92, 1.68)	0.0	2	0.74 (0.48, 1.13)	0.0
	Men, Women*	1	1.10 (0.08, 1.51)	–	1	1.19 (0.78, 1.81)	–	1	0.94 (0.57, 1.54)	–
Country	China	1	0.90 (0.64, 1.27)	–	1	1.10 (0.69, 1.76)	–	1	0.70 (0.39, 1.26)	–
	Japan	3	1.17 (0.97, 1.39)	4.8	3	1.35 (1.09, 1.66)	0.0	3	0.86 (0.64, 1.15)	0.0
	Korea	2	1.26 (1.01, 1.57)	82.0	–	–	–	–	–	–
	Singapore	–	–	–	–	–	–	–	–	–
No. participants	< 50,000	3	1.17 (0.97, 1.39)	4.8	1	1.19 (0.78, 1.81)	–	1	0.94 (0.57, 1.54)	–
	≥ 50,000	3	1.17 (0.95, 1.45)	75.7	3	1.33 (1.08, 1.65)	0.0	3	0.78 (0.57, 1.07)	0.0
*Fruits*										
Sex	Men	3	0.92 (0.70, 1.20)	74.3	2	0.96 (0.66, 1.39)	51.4	2	0.88 (0.38, 2.04)	84.8
	Women	2	1.01 (0.86, 1.20)	0.0	1	0.90 (0.63, 1.28)	–	1	0.90 (0.48, 1.69)	–
	Men, Women	1	0.89 (0.72, 1.10)	–	1	1.43 (0.75, 2.72)	–	1	1.33 (0.61, 2.90)	–
Country	China	1	0.67 (0.48, 0.94)	–	1	0.76 (0.49, 1.19)	–	1	0.56 (0.33, 0.96)	–
	Japan	4	1.01 (0.89, 1.15)	26.9	3	1.06 (0.86, 1.31)	0.0	3	1.21 (0.90, 1.63)	–
	Korea	–	–	–	–	–	–	–	–	–
	Singapore	1	0.89 (0.72, 1.10)	–	–	–	–	–	–	–
No. participants	< 50,000	2	1.05 (0.81, 1.37)	48.2	3	1.06 (0.86, 1.31)	0.0	3	1.21 (0.90, 1.63)	0.0
	≥ 50,000	4	0.91 (0.78, 1.05)	45.6	1	0.76 (0.49, 1.19)	–	1	0.56 (0.33, 0.96)	–
*Soy food*										
Sex	Men	1	0.89 (0.68, 1.17)	–	2	0.95 (0.60, 1.50)	61.5	1	1.20 (0.74, 1.95)	–
	Women	2	0.83 (0.54, 1.28)	74.4	3	0.80 (0.55, 1.17)	60.2	2	0.68 (0.42, 1.10)	37.5
	Men, Women	1	0.95 (0.78, 1.16)	–	–	–	–	–	–	–
Country	China	1	0.67 (0.49, 0.91)	–	1	0.76 (0.52, 1.12)	–	1	0.55 (0.34, 0.89)	–
	Japan	2	0.95 (0.78, 1.17)	0.0	4	0.88 (0.64, 1.22)	59.1	2	1.07 (0.73, 1.55)	0.0
	Korea	–	–	–	–	–	–	–	–	–
	Singapore	1	0.95 (0.78, 1.16)	–	–	–	–	–	–	–
No. participants	< 50,000	–	–	–	2	0.84 (0.38, 1.82)	80.4	–	–	–
	≥ 50,000	–	–	–	3	0.86 (0.68, 1.10)	25.8	3	0.84 (0.52, 1.35)	60.3
*Alcohol*										
Sex	Men	3	1.93 (1.00, 3.71)	86.7	1	3.44 (2.50, 4.72)	–	1	2.10 (1.16, 3.83)	–
	Women	3	1.49 (1.08, 2.07)	0.0	1	1.66 (1.12, 2.46)	–	1	2.39 (1.18, 4.88)	–
	Men, Women	2	1.48 (1.05, 2.07)	76.2	–	–	–	–	–	–
Country	China	2	1.03 (0.67, 1.59)	0.0	–	–	–	–	–	–
	Japan	2	2.18 (1.17, 4.05)	87.8	2	2.41 (1.18, 4.93)	87.5	2	2.22 (1.40, 3.50)	0.0
	Korea	3	1.51 (0.97, 2.34)	53.7	–	–	–	–	–	–
	Singapore	1	1.84 (1.31, 2.58)	–	–	–	–	–	–	–
No. participants	< 100,000	5	1.53 (1.07, 2.17)	39.9	–	–	–	–	–	–
	≥ 100,000	3	1.80 (1.05, 3.09)	94.8	2	2.41 (1.18, 4.93)	87.5	2	2.22 (1.40, 3.50)	0.0
*Coffee*										
Sex	Men	2	0.93 (0.84, 1.04)	0.0	3	0.90 (0.80, 1.03)	0.0	2	1.06 (0.89, 1.25)	0.0
	Women	2	1.07 (0.73, 1.58)	77.8	3	0.83 (0.49, 1.43)	81.2	2	1.18 (0.91, 1.52)	0.0
	Men, Women	–	–	–	1	0.90 (0.73, 1.11)	–	1	1.06 (0.82, 1.38)	–
Country	China	–	–	–	–	–	–	–	–	–
	Japan	2	0.91 (0.83, 1.00)	0.0	4	0.82 (0.67, 0.99)	41.8	2	1.08 (0.92, 1.26)	0.0
	Korea	2	1.16 (0.88, 1.53)	33.9	2	1.14 (0.73, 1.78)	58.7	2	1.17 (0.82, 1.69)	0.0
	Singapore	–	–	–	1	0.90 (0.73, 1.11)	–	1	1.06 (0.82, 1.38)	–
No. participants	< 100,000	–	–	–	3	0.75 (0.51, 1.10)	52.4	1	1.06 (0.82, 1.38)	–
	≥ 100,000	4	0.97 (0.85, 1.11)	41.4	4	0.94 (0.78, 1.13)	51.8	4	1.09 (0.95, 1.26)	0.0
*Fiber*										
Sex	Men	1	1.00 (0.84, 1.19)	–	–	–	–	–	–	–
	Women	2	1.05 (0.87, 1.26)	0.0	1	1.20 (0.60, 2.40)	–	1	0.90 (0.39, 2.06)	0.0
	Men, Women	2	0.91 (0.62, 1.31)	68.8	2	0.81 (0.44, 1.52)	83.7	2	1.03 (0.75, 1.42)	0.0
Country	China	1	1.10 (0.64, 1.91)	–	1	1.20 (0.60, 2.40)	–	1	0.90 (0.39, 2.06)	–
	Japan	3	0.96 (0.82, 1.13)	35.4	1	0.58 (0.38, 0.88)	–	1	1.10 (0.59, 2.06)	–
	Korea	–	–	–	–	–	–	–	–	–
	Singapore	1	1.07 (0.85, 1.34)	–	1	1.10 (0.83, 1.46)	–	1	1.01 (0.75, 1.37)	–
No. participants	< 50,000	1	0.73 (0.51, 1.04)	–	1	0.58 (0.38, 0.88)	–	1	1.10 (0.59, 2.06)	–
	≥ 50,000	4	1.03 (0.92, 1.15)	0.0	2	1.11 (0.86, 1.45)	0.0	2	0.99 (0.71, 1.39)	0.0
*Healthy pattern*										
Sex	Men	1	0.85 (0.72, 1.00)	–	1	0.85 (0.70, 1.04)	–	1	0.84 (0.63, 1.13)	–
	Women	1	0.97 (0.79, 1.19)	–	1	0.81 (0.64, 1.03)	–	1	1.53 (1.04, 2.23)	–
	Men, Women	2	0.89 (0.67, 1.18)	70.5	1	0.89 (0.66, 1.19)	–	1	0.56 (0.37, 0.84)	–
Country	China	–	–	–	–	–	–	–	–	–
	Japan	3	0.86 (0.76, 0.98)	15.2	3	0.85 (0.74, 0.97)	0.0	3	0.90 (0.53, 1.52)	84.4
	Korea	–	–	–	–	–	–	–	–	–
	Singapore	1	1.02 (0.83, 1.25)	–	–	–	–	–	–	–
No. participants	< 50,000	1	0.76 (0.60, 0.97)	–	1	0.89 (0.66, 1.20)	–	1	0.56 (0.37, 0.84)	0.0
	≥ 50,000	3	0.93 (0.83, 1.04)	6.0	2	0.83 (0.72, 0.97)	0.0	2	1.12 (0.62, 2.01)	83.3
*Unhealthy pattern*										
Sex	Men	1	0.90 (0.75, 1.07)	–	1	0.94 (0.76, 1.16)	–	1	0.82 (0.61, 1.10)	–
	Women	1	1.19 (0.95, 1.50)	–	1	1.28 (0.98, 1.68)	–	1	0.96 (0.61, 1.50)	–
	Men, Women	2	0.98 (0.84, 1.14)	16.5	1	0.87 (0.66, 1.13)	–	1	1.23 (0.86, 1.76)	–
Country	China	–	–	–	–	–	–	–	–	–
	Japan	3	1.01 (0.86, 1.18)	43.6	3	1.01 (0.81, 1.26)	56.4	3	0.97 (0.76, 1.25)	31.9
	Korea	–	–	–	–	–	–	–	–	–
	Singapore	1	0.97 (0.78, 1.20)	–	–	–	–	–	–	–
No. participants	< 50,000	1	0.99 (0.80, 1.23)	–	1	0.87 (0.66, 1.14)	0.0	1	1.23 (0.86, 1.76)	–
	≥ 50,000	3	1.00 (0.85, 1.17)	44.3	2	1.08 (0.80, 1.47)	68.0	2	0.86 (0.67, 1.10)	0.0

RR, relative risk; CI, confidence interval

^*^ ‘Men, Women’ indicates that the study results were analyzed for both sexes combined, without sex-specific stratification

A pooled RR of 1.93 (95% CI, 1.00–3.71, I^2^ = 86.7%) for men, 1.49 (95% CI, 1.08–2.07, I^2^ = 0.0%) for women, and 1.48 (95% CI, 1.05–2.07, I^2^ = 76.2%) for both sexes for CRC risk among the various studies investigating alcohol based on sex. Studies conducted in Japan and Singapore on CRC also demonstrated a significant risk associated with alcohol consumption, with pooled RRs of 2.18 (95% CI, 1.17–4.05, I^2^ = 87.8%) and 1.84 (95% CI, 1.31–2.58), respectively. In addition, the number of participants less than 100,000 had a pooled RR of 1.53 (95% CI, 1.07–2.17, I^2^ = 39.9%), whereas the number of participants greater than 100,000 had a pooled RR of 1.80 (95% CI, 1.05–3.09, I^2^ = 94.8%) for CRC. Meanwhile, studies conducted in Japan with more than 100,000 participants reported pooled RRs of 2.41 (95% CI, 1.18–4.93, I^2^ = 87.5%) for colon cancer and 2.22 (95% CI, 1.40–3.50, I^2^ = 0.0%) for rectal cancer.

### Publication bias

Only studies that assessed the associations of alcohol, green tea, coffee, total meat, red meat, processed meat, fish, fruits, vegetables, soy food, calcium, fiber, healthy patterns, and unhealthy patterns with CRC risk were assessed for publication bias due to the limited number of included studies. Egger’s and Begg’s tests confirmed the funnel plot asymmetry, detecting publication bias in the relationship between alcohol consumption and CRC risk (Egger’s test: *p* = 0.035, Begg’s test: *p*  = 0.217). However, there was no evidence of publication bias detected by the funnel plot. The funnel plot asymmetry was confirmed by Egger’s and Begg’s tests (green tea: Egger’s test: *p* = 0.691, Begg’s test: *p*  = 0.640; coffee: Egger’s test: *p* = 0.388, Begg’s test: *p* = 0.528; total meat: Egger’s test: *p* = 0.161, Begg’s test: *p* = 0.025; red meat: Egger’s test: *p* = 0.536, Begg’s test: *p* = 0.399; processed meat: Egger’s test: *p* = 0.810, Begg’s test: *p* = 0.807; fish: Egger’s test: *p* = 0.221, Begg’s test: *p* = 0.126; fruit: Egger’s test: *p* = 0.846, Begg’s test: *p* = 0.870; vegetables: Egger’s test: *p* = 0.761, Begg’s test: *p* = 0.547; soy food: Egger’s test: *p* = 0.599, Begg’s test: *p* = 0.784; calcium: Egger’s test: *p* = 0.064, Begg’s test: *p* = 0.392; fiber: Egger’s test: *p* = 0.529, Begg’s test: *p* = 0.484; healthy pattern: Egger’s test: *p* = 0.973, Begg’s test: *p* = 0.929; unhealthy pattern: Egger’s test: *p* = 0.510, Begg’s test: *p* = 0.421) (Supplementary Figure S2).

## Discussion

This systematic review and meta-analysis investigated the associations between various dietary factors and CRC risk in Asian populations. We observed that total meat, processed meat, and alcohol consumption increased CRC and colon cancer risk, whereas alcohol and white meat consumption increased the rectal cancer risk. We also found that calcium consumption may reduce CRC and colon cancer risk at borderline significance levels and that a healthy dietary pattern reduces colon cancer risk. While no significant results were found between CRC and other exposures, subgroup analyses revealed that sex, country, and the number of participants may influence the associations.

### Meat and fish consumption and CRC

Among the few studies that explored the overall effect of meat consumption on CRC risk, Parra-Soto et al. reported that meat eaters had a significantly increased risk of CRC compared with vegetarians in the UK biobank cohort [59]. Moreover, Woo et al. reported that meat increased the risk for CRC by 25% among Koreans [60], which aligns with our findings. Alternatively, Xu et al. did not observe significant associations in their meta-analyses based on Chinese cohorts [14]. The heterogeneity in the meat‒CRC risk association may stem from the meat types, consumption proportions; and the cooking methods, such as curing, smoking, or charring [61, 62].

We found that red meat consumption did not significantly affect the colorectal, colon, or rectal cancer risk. Previous meta-analyses among Asian populations have reported conflicting results. Di et al. pooled data from nine cohort studies on Asians and reported no significant association between the red meat quality and CRC risk. Pham et al. analyzed four Japanese cohort studies and reported a borderline association only with the colon cancer risk. In contrast, Wang et al. reported a significant association [63–65]. We found that white meat increased the rectal cancer risk at a borderline significance level. A previous meta-analysis by Shi et al. pooled one Australian and one Japanese cohort study and reported that white meat conferred a protective but not significant effect [66]. Our results may differ from those of Shi et al., as a more homogenous ethnic group of East Asians was used. Finally, our findings that processed meat increases CRC and colon cancer risk align with many the findings of previous meta-analyses [63, 65, 67].

Several mechanisms may link meat consumption to CRC risk. Red meat contains high levels of sulfur-containing amino acids, saturated fats, and heme iron, which can induce oxidative stress and promote the formation of carcinogenic N-nitroso compounds. Processing meat is thought to form mutagens, such as nitrates, nitrites, and polycyclic aromatic hydrocarbons [68]. Mechanistically, white meat consumption is believed to reduce CRC risk by substituting red meat [69]. However, the epidemiological evidence remains mixed, with some studies, including ours, showing disagreement, suggesting that factors such as absolute consumption and cooking methods may influence these associations.

No significant association between fish and CRC was found, which aligns with Pham et al. ’s cohort study meta-analysis [70]. Fish has been proposed to reduce CRC risk through a red meat substitution effect similar to white meat [69], although the evidence does not support this claim.

### Fruits, vegetables, soy, and dietary fiber intake and CRC

Although the evidence is limited, fruits and vegetables may reduce CRC risk [6, 71, 72]. Though not significant, our findings suggest that fruits, vegetables and fiber may be protective against CRC. Several mechanisms may explain this inverse association. Fiber increases stool bulk, shortens colon transit time, and dilutes carcinogens, potentially reducing colon cancer risk. Bioactive compounds such as flavonoids and glucosinolates may also prevent DNA damage and reduce oxidative stress [68, 73]. Among vegetables, soy has garnered attention in CRC research due to its content of isoflavones, plant sterols, and protease inhibitors—phytochemicals known for their potential anticancer properties, including DNA repair and antioxidant activity [74]. A previous meta-analysis of epidemiological studies by Yu et al. reported a protective effect of high soy consumption on CRC risk [75]. While our findings were not significant, a cohort study of Chinese women suggested a 33% reduction in CRC risk with soy consumption [32].

### Alcohol, green tea, coffee consumption and CRC

The association between alcohol consumption and CRC and colon and rectal cancer aligns with past literature, including several meta-analyses [14, 76, 77] as well as the WCRF/AICR 2018 report [6]. Alcohol intake is a risk factor for gastric, liver, and breast cancer, among others, and various oncogenic mechanisms have been suggested [78, 79]. For CRC, alcohol metabolites cause inflammation, immunosuppression, and DNA damage [80].

No significant associations were observed between green tea consumption and CRC risk. One meta-analysis by Chen et al. [81] and previous experimental studies have suggested polyphenols in green tea may inhibit heterocyclic aromatic amine-induced mutagenicity and decrease the risk of CRC [72], although epidemiological evidence does not support this claim [14, 82, 83]. Similar to green tea, caffeine and chlorogenic acids in coffee may confer a protective effect against CRC by encouraging apoptosis. Previous meta-analyses, however, failed to find a significant protective association between coffee and CRC [84–87]. While our findings are consistent with those of previous studies, our subgroup analyses revealed that the pooled results of Japanese studies indicate a borderline significant protective effect of coffee against CRC, suggesting the need for further research.

### Calcium intake and CRC

We found that calcium consumption decreases CRC and colon cancer risk. Previous pooled cohort studies and meta-analyses revealed that calcium has an inverse association with CRC risk in dietary and supplemental intake [88, 89]. Additionally, two RCTs reported that calcium supplementation decreased adenoma recurrence, suggesting the chemopreventive properties of calcium [90, 91]. Calcium may reduce the CRC risk through forming insoluble compounds with tumor-promoting free fatty acids and bile acids in the colon, potentially reducing their harmful effects [68, 92, 93].

### Dietary patterns and CRC

We observed that a healthy dietary pattern was associated with a significantly decreased risk of colon cancer, a result replicated in subgroups focused on studies conducted in Japan and studies with large sample sizes. Additionally, while not significant, these results suggested that healthy dietary patterns were protective against CRC and rectal cancer. A previous meta-analysis by Feng et al. revealed that healthy eating significantly decreased the CRC risk by 25% [94]. A healthy dietary pattern is characterized by consuming vegetables, fruits, lean proteins and a low intake of red and processed meats [95]. While no single mechanism explains the protective effects of healthy eating, we postulate that it is a combined effect of increased fiber consumption, plant-based bioactive compounds, phytochemicals, and reduced exposure to red- and processed-meat carcinogens via the replacement effect.

This meta-analysis has several strengths and a few limitations. We extensively searched literature on the associations between CRC risk and various dietary factors. We also explored the links between food groups and CRC risk in Asian populations, where research is limited despite the increasing incidence of CRC and differences in susceptibility, dietary habits, and food preparation methods. We performed extensive subgroup analyses by country, sex, and sample size to address heterogeneity. To minimize recall bias, we focused exclusively on cohort studies. However, high heterogeneity was observed in analyses of alcohol, meat, fruit, fiber, and healthy patterns, likely due to differences in study populations, food subtypes, and preparation methods. As such, we used a random-effects model and subgroup analyses to reduce heterogeneity. Moreover, a limitation of this review is the geographic scope of the included studies, which were limited to East Asian countries (Japan, China, Korea, Singapore and Taiwan). Although colorectal cancer is rising across Asia, no eligible cohort studies from South or Southeast Asia met our inclusion criteria. This likely reflects a lack of long-term, population-based research in those regions and underscores the need for future studies involving more diverse Asian populations.

In conclusion, we found that total meat, processed meat, and alcohol increased, whereas calcium and healthy dietary patterns decreased the risk of CRC. Our findings could help shape public dietary recommendations for CRC prevention in Asia. Further original research on Asian populations is needed to provide clearer region-specific insight into the dietary impacts on CRC risk.

## Supplementary Information

Below is the link to the electronic supplementary material.

Supplementary file1 (DOCX 822 KB)

## Data Availability

The datasets used and/or analyzed during the current study are available from the corresponding author on reasonable request.
