# Supplementary material for: Dietary factors and colorectal cancer risk in Asian populations: a systematic review and meta-analysis of cohort studies
Source: Cancer Causes Control. 2025 Aug 27;36(12):1647–61. doi: 10.1007/s10552-025-02053-9 (PMC12630220; doi:10.1007/s10552-025-02053-9)
Supplement: Supplementary file 1 — Supplementary file1 (DOCX 822 KB) [file 10552_2025_2053_MOESM1_ESM.docx]

**Supplementary Data**

**Appendix 1.** PRISMA checklist

**Appendix 2.** The Newcastle–Ottawa Scale

**Supplementary** **Table S1.** Search strategy

**Supplementary** **Table S2.** PICOS criteria for the inclusion of studies

**Supplementary** **Table S3.** Main characteristics of included studies that assessed the associations of food groups and dietary patterns with colorectal cancer risk

**Supplementary** **Table S4.** Quality assessment of included studies: adapted from the Newcastle‒Ottawa Scale

**Supplementary** **Table S5.** Summary of included cohort studies

**Supplementary Table S6.** Pooled results for food groups, dietary patterns and colorectal cancer risk

**Supplementary Figure S1.** Forest plots: associations between food groups, dietary patterns, and the incidence of colorectal, colon, and rectal cancer.

**Supplementary Figure S2.** Publication bias: associations between food groups, dietary patterns and the incidence of colorectal, colon, and rectal cancer.

*Appendix 1.* **PRISMA checklist**

| **Section and Topic** | **Item #** | **Checklist item** | **Location where item is reported.**  **PDF(Manuscript)** |
| --- | --- | --- | --- |
| **TITLE** | | |  |
| Title | 1 | Identify the report as a systematic review. | 1(1) |
| **ABSTRACT** | | |  |
| Abstract | 2 | See the PRISMA 2020 for Abstracts checklist. | 1(2) |
| **INTRODUCTION** | | |  |
| Rationale | 3 | Describe the rationale for the review in the context of existing knowledge. | 1(3) |
| Objectives | 4 | Provide an explicit statement of the objective(s) or question(s) the review addresses. | 2(3) |
| **METHODS** | | |  |
| Eligibility criteria | 5 | Specify the inclusion and exclusion criteria for the review and how studies were grouped for the syntheses. | 2(4) |
| Information sources | 6 | Specify all databases, registers, websites, organizations, reference lists and other sources searched or consulted to identify studies. Specify the date when each source was last searched or consulted. | 2(4) |
| Search strategy | 7 | Present the full search strategies for all databases, registers and websites, including any filters and limits used. | Supplementary Table S1 |
| Selection process | 8 | Specify the methods used to decide whether a study met the inclusion criteria of the review, including how many reviewers screened each record and each report retrieved, whether they worked independently, and if applicable, details of automation tools used in the process. | 2(4) |
| Data collection process | 9 | Specify the methods used to collect data from reports, including how many reviewers collected data from each report, whether they worked independently, any processes for obtaining or confirming data from study investigators, and if applicable, details of automation tools used in the process. | 2(4) |
| Data items | 10a | List and define all outcomes for which data were sought. Specify whether all results that were compatible with each outcome domain in each study were sought (e.g. for all measures, time points, analyses), and if not, the methods used to decide which results to collect. | 2(4) |
|  | 10b | List and define all other variables for which data were sought (e.g. participant and intervention characteristics, funding sources). Describe any assumptions made about any missing or unclear information. | 2(4) |
| Study risk of bias assessment | 11 | Specify the methods used to assess risk of bias in the included studies, including details of the tool(s) used, how many reviewers assessed each study and whether they worked independently, and if applicable, details of automation tools used in the process. | 2(4) |
| Effect measures | 12 | Specify for each outcome the effect measure(s) (e.g. risk ratio, mean difference) used in the synthesis or presentation of results. | 2(4) |
| Synthesis methods | 13a | Describe the processes used to decide which studies were eligible for each synthesis (e.g. tabulating the study intervention characteristics and comparing against the planned groups for each synthesis (item #5)). | 2(4) |
|  | 13b | Describe any methods required to prepare the data for presentation or synthesis, such as handling of missing summary statistics, or data conversions. | 2(4) |
|  | 13c | Describe any methods used to tabulate or visually display results of individual studies and syntheses. | 2(4) |
|  | 13d | Describe any methods used to synthesize results and provide a rationale for the choice(s). If meta-analysis was performed, describe the model(s), method(s) to identify the presence and extent of statistical heterogeneity, and software package(s) used. | 2(4) |
|  | 13e | Describe any methods used to explore possible causes of heterogeneity among study results (e.g. subgroup analysis, meta-regression). | 2(4) |
|  | 13f | Describe any sensitivity analyses conducted to assess robustness of the synthesized results. | 2(4) |
| Reporting bias assessment | 14 | Describe any methods used to assess risk of bias due to missing results in a synthesis (arising from reporting biases). | 2(4) |
| Certainty assessment | 15 | Describe any methods used to assess certainty (or confidence) in the body of evidence for an outcome. | 2(4) |
| **RESULTS** | | |  |
| Study selection | 16a | Describe the results of the search and selection process, from the number of records identified in the search to the number of studies included in the review, ideally using a flow diagram. | Fig. 1 |
|  | 16b | Cite studies that might appear to meet the inclusion criteria, but which were excluded, and explain why they were excluded. | Fig. 1 |
| Study characteristics | 17 | Cite each included study and present its characteristics. | Supplementary Table S3 |
| Risk of bias in studies | 18 | Present assessments of risk of bias for each included study. | Supplementary Table S4 |
| Results of individual studies | 19 | For all outcomes, present, for each study: (a) summary statistics for each group (where appropriate) and (b) an effect estimate and its precision (e.g. confidence/credible interval), ideally using structured tables or plots. | Supplementary Figure S1 |
| Results of syntheses | 20a | For each synthesis, briefly summarize the characteristics and risk of bias among contributing studies. | 3-7(5-15) |
|  | 20b | Present results of all statistical syntheses conducted. If meta-analysis was done, present for each the summary estimate and its precision (e.g. confidence/credible interval) and measures of statistical heterogeneity. If comparing groups, describe the direction of the effect. | 3-7(5-15), Fig. 2 |
|  | 20c | Present results of all investigations of possible causes of heterogeneity among study results. | 3-7(5-15), Fig. 2 |
|  | 20d | Present results of all sensitivity analyses conducted to assess the robustness of the synthesized results. | 7(14-15), Table 1 |
| Reporting biases | 21 | Present assessments of risk of bias due to missing results (arising from reporting biases) for each synthesis assessed. | None |
| Certainty of evidence | 22 | Present assessments of certainty (or confidence) in the body of evidence for each outcome assessed. | 3-7(5-15), Fig. 2 |
| **DISCUSSION** | | |  |
| Discussion | 23a | Provide a general interpretation of the results in the context of other evidence. | 9-11(16-18) |
|  | 23b | Discuss any limitations of the evidence included in the review. | 11(18-19) |
|  | 23c | Discuss any limitations of the review processes used. | 11(18-19) |
|  | 23d | Discuss implications of the results for practice, policy, and future research. | 11(18-19) |
| **OTHER INFORMATION** | | |  |
| Registration and protocol | 24a | Provide registration information for the review, including register name and registration number, or state that the review was not registered. | None |
|  | 24b | Indicate where the review protocol can be accessed, or state that a protocol was not prepared. | None |
|  | 24c | Describe and explain any amendments to information provided at registration or in the protocol. | None |
| Support | 25 | Describe sources of financial or non-financial support for the review, and the role of the funders or sponsors in the review. | None |
| Competing interests | 26 | Declare any competing interests of review authors. | 11(29) |
| Availability of data, code and other materials | 27 | Report which of the following are publicly available and where they can be found: template data collection forms; data extracted from included studies; data used for all analyses; analytic code; any other materials used in the review. | Appendix 1, Appendix 2 |

*From:* Page MJ, McKenzie JE, Bossuyt PM, Boutron I, Hoffmann TC, Mulrow CD, et al. The PRISMA 2020 statement: an updated guideline for reporting systematic reviews. BMJ 2021;372:n71. doi: 10.1136/bmj.n71. This work is licensed under CC BY 4.0. To view a copy of this license, visit <https://creativecommons.org/licenses/by/4.0/>

*Appendix 2.* **The Newcastle‒Ottawa Scale**

**NEWCASTLE−OTTAWA QUALITY ASSESSMENT SCALE**

**COHORT STUDIES**

Note: A study can be awarded a maximum of one star for each numbered item within the selection and outcome categories. A maximum of two stars can be given for comparability.

**Selection**

1) Representativeness of the exposed cohort

a) Truly representative of the underlying population **🟑**

b) Somewhat representative of the underlying population **🟑**

c) Selected groups of users, e.g., nurses, volunteers

d) No description of the derivation of the cohort

2) Selection of the non-exposed cohort

a) drawn from the same community as the exposed cohort **🟑**

b) drawn from a different source

c) no description of the derivation of the non-exposed cohort

3) Ascertainment of exposure

a) Secure record (e.g., surgical records) **🟑**

b) Structured interview **🟑**

c) Written self-reports

d) No description

4) Demonstration that the outcome of interest was not present at the start of the study

a) Yes **🟑**

b) No

**Comparability**

1) Comparability of cohorts on the basis of their design or analysis

a) Study controls for age and sex **🟑**

b) Study controls for any additional factor **🟑**

**Outcome**

1) Assessment of outcomes

a) Independent blind assessment **🟑**

b) Record linkage **🟑**

c) Self-report

d) No description

2) Was follow-up long enough for outcomes to occur?

a) Yes (follow-up ≥5 years) **🟑**

b) Mo

3) Adequacy of follow-up of cohorts

a) Complete follow-up; all subjects accounted for **🟑**

b) Subjects lost to follow-up unlikely to introduce bias; small number lost, <20% (select an adequate %) follow-up, or description provided of those lost) **🟑**

c) Follow-up rate <80% (select an adequate %) and no description of those lost

d) No statement

**Supplementary** **Table S1***.* Search strategy

|  | | Presearch term | Pubmed | Embase | Web of Science | SCOPUS |
| --- | --- | --- | --- | --- | --- | --- |
| Exposure | #1 | food | [MeSH Terms] OR [Title/Abstract] | /exp OR :ti,ab | ti, ab, key | ti, ab, key |
|  | #2 | beverages | [MeSH Terms] OR [Title/Abstract] | /exp OR :ti,ab | ti, ab, key | ti, ab, key |
|  | #3 | diet | [MeSH Terms] OR [Title/Abstract] | /exp OR :ti,ab | ti, ab, key | ti, ab, key |
|  | #4 | nutrient | [MeSH Terms] OR [Title/Abstract] | /exp OR :ti,ab | ti, ab, key | ti, ab, key |
|  | #5 | intake | [Title/Abstract] | :ti,ab | ti, ab, key | ti, ab, key |
|  | #6 | consumption | [Title/Abstract] | :ti,ab | ti, ab, key | ti, ab, key |
|  | #7 | #1 - #6 combined with 'OR' | | | | |
| Outcome | #8 | colorectal neoplasms | [MeSH Terms] OR [Title/Abstract] | /exp OR :ti,ab | ti, ab, key | ti, ab, key |
|  | #9 | colorectal | [Title/Abstract] | :ti,ab | ti, ab, key | ti, ab, key |
|  | #10 | colon | [MeSH Terms] OR [Title/Abstract] | /exp OR :ti,ab | ti, ab, key | ti, ab, key |
|  | #11 | rectal | [MeSH Terms] OR [Title/Abstract] | /exp OR :ti,ab | ti, ab, key | ti, ab, key |
|  | #12 | colon | [Title/Abstract] | :ti,ab | ti, ab, key | ti, ab, key |
|  | #13 | rectum | [MeSH Terms] OR [Title/Abstract] | /exp OR :ti,ab | ti, ab, key | ti, ab, key |
|  | #14 | bowel | [Title/Abstract] | :ti,ab | ti, ab, key | ti, ab, key |
|  | #15 | #9 - #14 combined with 'OR' | | | | |
|  | #16 | cancer | [Title/Abstract] | :ti,ab | ti, ab, key | ti, ab, key |
|  | #17 | neoplasm | [MeSH Terms] OR [Title/Abstract] | /exp OR :ti,ab | ti, ab, key | ti, ab, key |
|  | #18 | adenoma | [MeSH Terms] OR [Title/Abstract] | /exp OR :ti,ab | ti, ab, key | ti, ab, key |
|  | #19 | tumor, tumour | [MeSH Terms] OR [Title/Abstract] | /exp OR :ti,ab | ti, ab, key | ti, ab, key |
|  | #20 | carcinoma | [MeSH Terms] OR [Title/Abstract] | /exp OR :ti,ab | ti, ab, key | ti, ab, key |
|  | #21 | #16 - #20 combined with 'OR' | | | | |
|  | #22 | #8 OR (#15 AND #21) | | | | |
| Total | #23 | #7 AND #22 | | | | |

**Supplementary Table S2.** PICOS criteria for the inclusion of studies

| Parameter | Description |
| --- | --- |
| Population | Healthy adults |
| Intervention | Highest consumption of food groups, nutrients or better adherence to dietary pattern |
| Comparison | Lowest consumption of food groups, nutrients or lower adherence to dietary pattern |
| Outcomes | Colorectal cancer, Colon cancer, Rectal cancer incidence |
| Study design | Cohort study |

**Supplementary Table S3.** Main characteristics of the included studies that assessed the associations of food groups and dietary patterns with colorectal cancer risk

| **Author and Year** | **Study** | **Age, years** | **Follow-up, years** | **Cases/Participants** | **Men (%)** | **Exposure variables** | **Comparisons** | **NOS** | **Country** |
| --- | --- | --- | --- | --- | --- | --- | --- | --- | --- |
| **JPHC Study** |  |  |  |  |  |  |  |  |  |
| Otani et al., 2003 [1] | JPHC | 40-69 | JPHC I: 10 | 716/90,004 | 47 | Alcohol | Regular drinkers (≥300 g/week) vs. Never drinkers | High | Japan |
|  |  |  | JPHC II: 7 |  |  |  |  |  |  |
| Kobayashi et al., 2004 [2] | JPHC | 40-69 | (max) 10 | 764/88,658 | 48 | Total fish, fatty acids | Total fish: almost daily vs. almost never | High | Japan |
| Tsubono et al., 2005 [3] | JPHC I | 40-59 | 694,074 PY | 705/40,106 | 48 | Fruits and vegetables | Q4 vs. Q1 | High | Japan |
|  | JPHC II | 40-69 |  | -/48,552 | 48 |  |  | High |  |
| Kim et al., 2005 [4] | JPHC I | 40-59 | 10 | 370/42,112 | 48 | Factor analysis - 3 patterns (Western/Traditional/Healthy) | Q4 vs. Q1 | High | Japan |
| Otani et al., 2006 [5] | JPHC | 40-69 | 10 | 907/86,412 | 47.2 | Dietary fiber | (M) 18.7 g/day vs. 6.4 g/day | High | Japan |
|  |  |  |  |  |  |  | (W) 20.0 g/day vs. 8.3 g/day |  |  |
| Lee et al., 2007 [6] | JPHC | 51.2 | (mean) 10 | 1,163/96,162 | 48 | Coffee, green tea | 3+ cups vs. Almost never | High | Japan |
| Ishihara et al., 2007 [7] | JPHC | 45-74 | (mean) 5.8 | 526/81,184 | 47 | Folate, vitamin B6, vitamin B12, methionine | Q4 vs. Q1 | High | Japan |
| Akhter et al., 2008 [8] | JPHC | 45-74 | (mean) 7.6 | 886/83,063 | 47 | Isoflavone, miso soup, soy food | Highest vs. Lowest | High | Japan |
| Ishihara et al., 2008 [9] | JPHC | 45-74 | (mean) 7.8 | 761/74,639 | 47 | Calcium, vitamin D | Dietary calcium: >661 mg/day vs. <337 mg/day | High | Japan |
| Ma et al., 2010 [10] | JPHC | 45-74 | 7.9 | 1,129/87,117 | 47 | Magnesium | Q5 (Low) vs. Q1 (High) | High | Japan |
| Takachi et al., 2010 [11] | JPHC | 40-69 | 598,763 PY | 836/77,500 | 54 | Sodium, salted foods | Q5 vs. Q1 | High | Japan |
| Takachi et al., 2011 [12] | JPHC | 45-74 | 758,116 PY | 1,145/80,658 | 48 | Total meat, red meat, beef, pork, processed meat, chicken | Total meat: Q5 (117 g/day) vs. Q1 (20 g/day) | High | Japan |
| Sasazuki et al., 2011 [13] | JPHC | 40-69 | (mean) 9.3 | 396/88,574 | - | Fatty acids | Q5 vs. Q1 | High | Japan |
| Hara et al., 2012 [14] | JPHC | 45-74 | 808,053 PY | 1,284/85,097 | 47 | Zinc, heme iron | Q4 vs. Q1 | High | Japan |
| Abe et al., 2014 [15] | JPHC | 40-69 | 11 | 1,276/73,501 | 47 | Rice, bread, noodles, cereal | Q4 vs. Q1 | High | Japan |
| Abe et al., 2016 [16] | JPHC | 40-69 | (mean) 12.5 | 1,468/73,501 | 47 | Glycemic load, glycemic index | Q4 vs. Q1 | High | Japan |
| Shin et al., 2018 [17] | JPHC | 40-69 | (mean) 13.8 | 2,482/93,062 | 47 | Factor analysis - 3 patterns (Western/Traditional/Prudent) | Q5 vs. Q1 | High | Japan |
| Mori et al., 2019 [18] | JPHC | 40-69 | (median) 15.9 | 2,612/88,172 | 47 | Cruciferous vegetables | Q4 vs. Q1 | High | Japan |
| Leung et al., 2021 [19] | JPHC | 45-74 | (mean) 14.7 | 1,648/74,070 | 46 | Sugary drink | ≥254 mL/d vs. Non consumers | High | Japan |
| Cai et al., 2022 [20] | JPHC | 45-74 | (median) 17.0 | 1,650/90,171 | - | Low-carbohydrate diet | Q5 vs. Q1 | High | Japan |
| Kanehara et al., 2023 [21] | JPHC | 45-69 | (mean) 15.0 | 2,118/91,005 | 47 | Total sugars, total fructose | Q5 vs. Q1 | High | Japan |
| Hori et al., 2023 [22] | JPHC | 40-70 | (mean) 15 | 2,261/89,283 | 47 | Fruits and vegetables, green and yellow vegetable, total fruit, citrus fruit, vitamin C, fiber, beta-carotene | Highest vs. Lowest | High | Japan |
| **JACC Study** |  |  |  |  |  |  |  |  |  |
| Wakai et al., 2005 [23] | JACC | 40-79 | 7.6 | 629/57,736 | 41 | Alcohol | (M) 3.0+ drinks/day vs. Nondrinkers | High | Japan |
|  |  |  |  |  |  |  | (W) 1.0+ drinks/day vs. Nondrinkers |  |  |
| Wakai et al., 2007 [24] | JACC | 40-79 | 7.6 | 443/43,115 | 38.6 | Dietary fiber, insoluble dietary fiber, fruit fiber, vegetable fiber, bean fiber | (M) 13.4±3.0 g/day vs. 6.7±2.0 g/day | High | Japan |
|  |  |  |  |  |  |  | (W) 13.4±2.8 g/day vs. 7.4±2.1 g/day |  |  |
| Aoyama et al., 2014 [25] | JACC | 40-79 | 598,605 PY | 806/45,516 | 42 | Fruits and vegetables | Middle & high vs. Low | High | Japan |
| Yamada et al., 2014 [26] | JACC | 40-79 | 738,669 PY | 687/58,221 | 41 | Coffee | ≥4 cups/day vs. <1 cup/day | High | Japan |
| Kato et al., 2023 [27] | JACC | 40-79 | (median) 13.8 | 876/42,536 | - | Fatty acids | Q4 vs. Q1 | High | Japan |
| **Takayama Study** |  |  |  |  |  |  |  |  |  |
| Shimizu et al., 2003 [28] | Takayama | ≥35 | 8 | 295/29,051 | 46 | Alcohol | >36.7 g/day vs. No alcohol | High | Japan |
| Oba et al., 2006 [29] | Takayama | ≥35 | 8 | 213/30,221 | 46 | Total energy, total fat, SFA, MUFA, PUFA, long n-3 FA, total protein, total meat, red meat, processed meat, coffee, green tea | 1 cup/day or more vs. Never to <1 cup/day | High | Japan |
| Oba et al., 2007 [30] | Takayama | ≥35 | 8 | 213/30,221 | 46 | Soy products, isoflavones | Highest vs. Lowest | High | Japan |
| Wada et al., 2017 [31] | Takayama | ≥35 | (max) 16 | 772/30,331 | 46 | Total meat, red meat, processed meat | Q4 vs. Q1 | High | Japan |
| Wada et al., 2019 [32] | Takayama | ≥35 | (max) 16 | 772/30,331 | 46 | Green tea | ≥4 times per day vs. <Once per day | High | Japan |
| Wada et al., 2022 [33] | Takayama | ≥35 | (mean) 13.3 | 712/30,722 | 46 | Nε-­carboxymethyl-­lysine | Q4 vs. Q1 | High | Japan |
| **Miyagi Study** |  |  |  |  |  |  |  |  |  |
| Nakaya et al., 2005 [34] | Miyagi | 40-64 | (max) 8 | 173/21,201 | 100 | Alcohol | ≥22.8 g/day vs. Never-drinkers | High | Japan |
| Suzuki et al., 2005 [35] | Miyagi 1 | ≥40 | 7 to 9 | 269/26,311 | - | Green tea | >5 cups/day vs. <1 cup/day | High | Japan |
|  | Miyagi 2 | 40-64 | 7 to 9 | 247/39,604 | - |  |  |  |  |
| Sato et al., 2005 [36] | Miyagi | 40-64 | 7 | 275/41,835 | 48 | Fruits and vegetables | Q4 vs. Q1 | High | Japan |
| Sato et al., 2006 [37] | Miyagi |  | 11 | 474/41,835 | 50 | Total meat, beef, pork, ham/sausage, chicken, liver | Total meat: Q4 (70.4 g/day) vs. Q1 (40.3 g/day) | High | Japan |
|  |  |  |  |  |  |  | Beef 1-2 times/week vs. Almost never |  |  |
|  |  |  |  |  |  |  | Pork: 1-2 times/week vs. Almost never |  |  |
|  |  |  |  |  |  |  | Ham/sausage: 1-2 times/week vs. Almost never |  |  |
|  |  |  |  |  |  |  | Chicken: 1-2 times/week vs. Almost never |  |  |
| Naganuma et al., 2007 [38] | Miyagi | 40-64 | 11.6 | 457/38,701 | 59 | Coffee | ≥3 cups/day vs. Never | High | Japan |
| Akhter et al., 2007 [39] | Miyagi | 40-64 | 11 | 307/21,199 | 100 | Alcohol | Heavy drinkers (>45.6 g/day) vs. Never drinkers | High | Japan |
| **Ohsaki Study** |  |  |  |  |  |  |  |  |  |
| Sugawara et al., 2009 [40] | Ohsaki | 40-79 | 9 | 566/39,498 | - | Total fish | Total fish: ≥96.40 g/day vs. 0–26.2 g/day | High | Japan |
| Li et al., 2010 [41] | Ohsaki | 40-79 | 9 | 665/42,470 | - | Citrus fruits | Daily vs. ≤2 times/week | High | Japan |
| Kumagai et al., 2014 [42] | Ohsaki | 40-79 | 11 | 854/44,097 | 48 | Factor analysis - 3 patterns (Animal food/Japanese/Dairy) | Q4 vs. Q1 | High | Japan |
| **Pooled studies of Japanese cohort studies** | | |  |  |  |  |  |  |  |
| Mizoue et al., 2008 [43] | JPHC I | 40-59 | (mean) 13.5 | 694/41,159 | 48 | Alcohol | (M) Current drinkers (>92 g/day) vs. Nondrinkers | High | Japan |
|  | JPHC II | 40-69 | (mean) 10.5 | 781/59,067 | 46 | Alcohol | (W) Current drinkers (>23 g/day) vs. Nondrinkers | High |  |
|  | JACC | 40-79 | (mean) 10.4 | 562/39,999 | 41 | Alcohol |  | High |  |
|  | Miyagi | 40-64 | (mean) 11.0 | 482/38,783 | 53 | Alcohol |  | High |  |
|  | Takayama | ≥35 | (mean) 6.9 | 283/30,755 | 46 | Alcohol |  | High |  |
| Kashino et al., 2018 [44] | JPHC I | 40-59 | (mean) 20.7 | 1,432/42,141 | 48 | Coffee | ≥3 cups/day vs. <1 cup/day | High | Japan |
|  | JPHC II | 40-69 | 17.1 | 1,685/61,779 | 47 |  |  |  |  |
|  | JACC | 40-79 | 13.1 | 1,032/53,990 | 41 |  |  |  |  |
|  | Miyagi | 40-64 | 15.8 | 817/38,645 | 49 |  |  |  |  |
|  | Ohsaki | 40-79 | 9 | 675/39,071 | 49 |  |  |  |  |
|  | 3-pref Miyagi | ≥40 | 7.5 | 227/22,404 | 47 |  |  |  |  |
|  | 3-pref Aichi | ≥40 | 11.5 | 409/30,338 | 48 |  |  |  |  |
|  | 3-pref Osaka | ≥40 | 12.1 | 434/31,954 | 47 |  |  |  |  |
| Islam et al., 2019 [45] | JPHC I | 40-59 | (median) 20 | 1,377/40,965 | 48 | Beef, pork, processed meat, chicken | Almost every day vs. <1 time/week | High | Japan |
|  | JPHC II | 40-69 | (median) 16.6 | 1,538/57,740 | 48 |  |  |  |  |
|  | JACC | 40-79 | (median) 15.6 | 1,313/70,602 | 41 |  |  |  |  |
|  | Miyagi | 40-64 | (median) 16.1 | 743/31,613 | 50 |  |  |  |  |
|  | Ohsaki | 40-79 | (median) 10.9 | 723/31,483 | 49 |  |  |  |  |
|  | JPHC | 44-76 | (median) 15 | 2,749/92,836 | 47 |  |  |  |  |
|  | Takayama | ≥40 | (median) 13.5 | 801/30,799 | 46 |  |  |  |  |
| **Jiashan Study** |  |  |  |  |  |  |  |  |  |
| Chen et al., 2005 [46] | Jiashan | ≥30 | 10.6 | 242/64,100 | 48 | Alcohol | Daily drinkers vs. Non-drinkers | High | China |
| **CKB Study** |  |  |  |  |  |  |  |  |  |
| Li et al., 2019 [47] | CKB | 30-79 | (median) 10.1 | 2,267/455,981 | 36 | Tea | Daily vs. Less than weekly | High | China |
| Im et al., 2021 [48] | CKB | 30-79 | 10 | 1,527/209,237 | 100 | Alcohol | Current regular drinkers (280 g/week) vs. Abstainers | High | China |
| Chan et al., 2021 [49] | CKB | 30-79 | (median) 10.1 | 3,061/510,101 | 44 | Spicy food | 6–7 days/week vs. Never/rarely | High | China |
| Kakkoura et al., 2022 [50] | CKB | 30-79 | (mean) 10.8 | 3,350/510,146 | 41 | Dairy food |  | High | China |
| **SWHS Study** |  |  |  |  |  |  |  |  |  |
| Shin et al., 2006 [51] | SWHS | 40-70 | (median) 5.74 | 283/73,314 | 0 | Calcium, fiber, vitamin A, retinol, carotene, thiamine, riboflavin, niacin, ascorbic acid, vitamin E | Dietary calcium: >610.8 mg/day vs. ≤291.9 mg/day | High | China |
| Yang et al., 2007 [52] | SWHS | 40-70 | 6 | 256/69,710 | 0 | Green tea | 150 g/month vs. 50 g/month | High | China |
| Shrubsole et al., 2009 [53] | SWHS | 40-70 | - | 394/72,861 | 0 | Folate, vitamin B6, B12, niacin, riboflavin, methionine | Q5 vs. Q1 | High | China |
| Murff et al., 2009 [54] | SWHS | 40-70 | (max) 12 | 961/73,242 | - | Total fish, Fatty acids | Total fish: 66.0 g/day vs. 21.1 g/day | High | China |
| Yang et al., 2009 [55] | SWHS | 40-70 | (mean) 6.4 | 321/68,412 | 0 | Soy foods, soy protein, isoflavones | T3 vs. T1 | High | China |
| Lee et al., 2009 [56] | SWHS | 40-70 | 7.4 | 394/73,224 | 0 | Total meat, red meat, white meat, total fish, marine fish, freshwater fish, eel, shrimp, shellfish, total fat, SFA, MUFA, PUFA, cholesterol | Q4 vs. Q1 | High | China |
| Li et al., 2011 [57] | SWHS | 40-70 | 9.1 | 475/73,061 | 0 | Glycemic load, glycemic index, carbohydrates | Q5 vs. Q1 | High | China |
| Nechuta et al., 2012 [58] | SWHS | 40-70 | (mean) 11 | 586/69,310 | 0 | Tea (any tea, green tea) | 150 g vs. 0 g | High | China |
| Pradhan et al., 2023 [59] | SWHS | 40-70 | (mean) 14.7 | 875/65,732 | 0 | Ginseng | >500 g vs. <500 g | High | China |
| **SMHS Study** |  |  |  |  |  |  |  |  |  |
| Yang et al., 2011 [60] | SMHS | 40-74 | (mean) 4.6 | 243/60,567 | 100 | Green tea | 425 g vs. 150 g | High | China |
| Vogtmann et al., 2013 [61] | SMHS | 40-74 | (median) 6.3 | 398/61,274 | 100 | Fruits and vegetables | Total fruits and vegetables: 675.15 g/day vs. <284.34 g/day | High | China |
|  |  |  |  |  |  |  | Total vegetables: 466.64 g/day vs. <192.60 g/day |  |  |
|  |  |  |  |  |  |  | Total fruits: 239.24 g/day vs. <42.38 g/day |  |  |
| Nguyen et al., 2020 [62] | SWHS | 40-74 | (mean) 13.4 | 979/72,445 | 0 | Chinese Food Pagoda Score | Q4 vs. Q1 | High | China |
|  | SMHS | 40-74 | (mean) 8.1 | 691/60,161 | 100 |  |  |  |  |
| Nguyen et al., 2021 [63] | SMHS | 40-74 | (mean) 9.8 | 1,268/59,986 | 100 | Fatty acids | Q4 vs. Q1 | High | China |
| **Pooled study of Japanese and Chinese cohort studies** | | |  |  |  |  |  |  |  |
| Khankari et al., 2020 [64] | SMHS | - | 12.7 | 561/56,786 | 100 | Soy isoflavone, soy protein | Q4 vs. Q1 | High | China, Japan |
|  | SWHS | - |  | 765/67,842 | 0 |  |  |  |  |
|  | JPHC I | - |  | 796/35,732 | 48 |  |  |  |  |
|  | JPHC II | - |  | 849/44,700 | 48 |  |  |  |  |
| **NHIS Study** |  |  |  |  |  |  |  |  |  |
| Kim et al., 2011 [65] | NHIS | 30-80 | 7 | 6,444/2,248,129 | 63 | Total meat | Total meat: ≥ 4 times/week vs. ≤ once/week | High | Korea |
| Choi et al., 2017 [66] | NHIS | >20 | (median) 5.4 | 154,970/23,179,302 | 51 | Alcohol | Heavy drinkers (30+ g/day) vs. Nondrinkers | High | Korea |
| Yoo et al., 2021 [67] | NHIS | ≥40 | 6.4 | 319,202/11,737,467 | 48 | Alcohol | Heavy drinker (≥210 g/week) vs. Nondrinker | High | Korea |
| Bui et al., 2022 [68] | NHIS | - | 10.5 | 26,887/2,839,332 | 100 | Alcohol | ≥50 g/day vs. 0 g/day | High | Korea |
| Yoo et al., 2022 [69] | NHIS | ≥40 | 6.4 | 80,263 alcohol-related cancers/4,513,746 | 51 | Alcohol | Changes in alcohol consumption | High | Korea |
|  |  |  |  |  |  |  | (nondrinker, sustainer, increaser, quitter, reducer) |  |  |
| **HEXA Study** |  |  |  |  |  |  |  |  |  |
| Lee et al., 2021 [70] | HEXA | 40-69 | (mean) 5.4 | 635/119,501 | 34 | Calcium | Dietary calcium: 200 g/day vs. <Recommended intake of calcium | High | Korea |
| Na et al., 2022 [71] | HEXA | 40-79 | (max) 15 | 928/114,243 | 34 | Coffee, Green tea | ≥ 2 cups vs. None | High | Korea |
| **CSEC Study** |  |  |  |  |  |  |  |  |  |
| Wie et al., 2014 [72] | CSEC | - | (median) 7 | 53/8,071 | 55 | Red meats, vegetables and fruits, Sodium | Red meat: ≥43 g/day vs. <43 g/day | High | Korea |
| **KMCC Study** |  |  |  |  |  |  |  |  |  |
| Cho et al., 2015 [73] | KMCC | >20 | (median) 11.2 | 220/18,522 | 40 | Alcohol | ≥30 g/day vs. Never drinkers | High | Korea |
| **SCHS Study** |  |  |  |  |  |  |  |  |  |
| Tsong et al., 2007 [74] | SCHS | 45-74 | (mean) 8.9 | 845/61,321 | . | Alcohol | 7+ drinks/week vs. Nondrinkers | High | Singapore |
| Sun et al., 2007 [75] | SCHS | 45-74 | (mean) 8.9 | 845/61,320 | 55 | Green tea, black tea | Daily vs. Non tea drinkers | High | Singapore |
| Butler et al., 2008 [76] | SCHS | 45-74 | (mean) 9.8 | 961/61,321 | - | Factor analysis – 2 patterns (Meat/Vegetable), fiber, micronutrients, soy foods, meat, fish | Q4 vs. Q1 | High | Singapore |
| Butler et al., 2009 [77] | SCHS | 45-74 | (mean) 9.8 | 213/61,321 | 45 | Total fat, fatty acids | Q4 vs. Q1 | High | Singapore |
| Peterson et al., 2010 [78] | SCHS | 45-74 | (mean) 9.8 | 961/61,321 | - | Coffee | 2 cups/day or more vs. <1 cup/day | High | Singapore |
| Yu et al., 2022 [79] | SCHS | 45-74 | (mean) 17.5 | 2,140/61,321 | 56 | Fiber, nonstarch polysaccharide | Q4 vs. Q1 | High | Singapore |
| Yu et al., 2022 [80] | SCHS | 45-74 | (mean) 17.5 | 2,140/61,321 | 56 | Composite dietary antioxidant index | Q4 vs. Q1 | High | Singapore |
| Yu et al., 2023 [81] | SCHS | 45-74 | (mean) 19.5 | 2,520/61,321 | - | Low-carbohydrate diet | Q4 vs. Q1 | High | Singapore |
| **Taiwanese Study** |  |  |  |  |  |  |  |  |  |
| Yeh et al., 2006 [82] | - | 30-65 | 10 | 107/22,115 | 54 | Peanut products, sweet potato, bean products, pickled foodstuffs, nitrated foodstuffs, smoked foodstuffs | Bean and peanut: 2 or more meals/week vs. 0-1 meal/week | High | Taiwan |

NOS: Newcastle‒Ottawa Scale; JPHC: Japan Public Health Center; g: grams; vs.: versus; max: maximum; PY: person-year; Q: Quintile; M: Men; W: Women; JACC: the Japan Collaborative Cohort Study; SFA: saturated fatty acids; MUFA: monounsaturated fatty acids; PUFA: polyunsaturated fatty acids; long n-3 FA: long-chain omega-3 fatty acids; Takayama: the Takayama Cohort Study; Nε-carboxymethyl-lysine: an advanced glycation end product; Miyagi: the Miyagi Cohort Study; Ohsaki: the Ohsaki National Health Insurance Cohort Study; 3-pref Miyagi: the Three Prefecture Study–Miyagi portion; 3-pref Aichi: the Three Prefecture Study–Aichi portion; 3-pref Osaka: the Three Prefecture Study–Osaka portion; Jiashan study: population-based cohort study in Jiashan County; CKB: the China Kadoorie Biobank; SWHS: the Shanghai Women’s Health Study; SMHS: the Shanghai Men’s Health Study; NHIS: the National Health Insurance System; HEXA: the Health Examinees Study; CSEC: the Cancer Screening Examination Cohort; KMCC: The Korean Multi-Center Cancer Cohort; SCHS: the Singapore Chinese Health Study;

**Supplementary Table S4.** Quality assessment of included studies: adapted from the Newcastle‒Ottawa Scale

| **Ref #** | **First author** | **Published year** | **Selection**  **(maximum 4*)** | | | | **Comparability**  **(maximum 2*)** | **Outcome**  **(maximum 3*)** | | | **Total**  **Score (0-9)** |
| --- | --- | --- | --- | --- | --- | --- | --- | --- | --- | --- | --- |
|  |  |  | 1) | 2) | 3) | 4) | 1) | 1) | 2) | 3) |  |
|  |  |  | Representativeness of the exposed cohort | Selection of the nonexposed cohort | Ascertainment of exposure | Demonstration that outcome of interest was not present at start of study | Comparability of cohorts on the basis of the design or analysis | Assessment of outcome | Was follow-up long enough for outcomes to occur? | Adequacy of follow-up of cohorts |  |
| **JPHC Study** |  |  |  |  |  |  |  |  |  |  |  |
| 1 | Otani | 2003 | * | * | * | * | ** | * | * |  | 8 |
| 2 | Kobayashi | 2004 | * | * | * | * | ** | * | * |  | 8 |
| 3 | Tsubono | 2005 | * | * | * | * | ** | * | * |  | 8 |
| 4 | Kim | 2005 | * | * | * | * | ** | * | * |  | 8 |
| 5 | Otani | 2006 | * | * | * | * | ** | * | * |  | 8 |
| 6 | Lee | 2007 | * | * | * | * | ** | * | * |  | 8 |
| 7 | Ishihara | 2007 | * | * | * | * | ** | * | * |  | 8 |
| 8 | Akhter | 2008 | * | * | * | * | ** | * | * |  | 8 |
| 9 | Ishihara | 2008 | * | * | * | * | ** | * | * |  | 8 |
| 10 | Ma | 2010 | * | * | * | * | ** | * | * |  | 8 |
| 11 | Takachi | 2010 | * | * | * | * | ** | * | * |  | 8 |
| 12 | Takachi | 2011 | * | * | * | * | ** | * | * |  | 8 |
| 13 | Sasazuki | 2011 | * | * | * | * | ** | * | * |  | 8 |
| 14 | Hara | 2012 | * | * | * | * | ** | * | * |  | 8 |
| 15 | Abe | 2014 | * | * | * | * | ** | * | * |  | 8 |
| 16 | Abe | 2016 | * | * | * | * | ** | * | * |  | 8 |
| 17 | Shin | 2018 | * | * | * | * | ** | * | * |  | 8 |
| 18 | Mori | 2019 | * | * | * | * | ** | * | * |  | 8 |
| 19 | Leung | 2021 | * | * | * | * | ** | * | * |  | 8 |
| 20 | Cai | 2022 | * | * | * | * | ** | * | * |  | 8 |
| 21 | Kanehara | 2023 | * | * | * | * | ** | * | * |  | 8 |
| 22 | Hori | 2023 | * | * | * | * | ** | * | * |  | 8 |
| **JACC Study** |  |  |  |  |  |  |  |  |  |  |  |
| 23 | Wakai | 2005 | * | * | * | * | ** | * | * |  | 8 |
| 24 | Wakai | 2007 | * | * | * | * | ** | * | * |  | 8 |
| 25 | Aoyama | 2014 | * | * | * | * | ** | * | * |  | 8 |
| 26 | Yamada | 2014 | * | * | * | * | ** | * | * |  | 8 |
| 27 | Kato | 2023 | * | * | * | * | ** | * | * |  | 8 |
| **Takayama Study** |  |  |  |  |  |  |  |  |  |  |  |
| 28 | Shimizu | 2003 | * | * | * | * | ** | * | * | * | 9 |
| 29 | Oba | 2006 | * | * | * | * | ** | * | * | * | 9 |
| 30 | Oba | 2007 | * | * | * | * | ** | * | * | * | 9 |
| 31 | Wada | 2017 | * | * | * | * | ** | * | * | * | 9 |
| 32 | Wada | 2019 | * | * | * | * | ** | * | * | * | 9 |
| 33 | Wada | 2022 | * | * | * | * | ** | * | * | * | 9 |
| **Miyagi Study** | |  |  |  |  |  |  |  |  |  |  |
| 34 | Nakaya | 2005 | * | * | * | * | ** | * | * | * | 9 |
| 35 | Suzuki | 2005 | * | * | * | * | ** | * | * | * | 9 |
| 36 | Sato | 2005 | * | * | * | * | ** | * | * | * | 9 |
| 37 | Sato | 2006 | * | * | * | * | ** | * | * | * | 9 |
| 38 | Naganuma | 2007 | * | * | * | * | ** | * | * | * | 9 |
| **Ohsaki Study** | |  |  |  |  |  |  |  |  |  |  |
| 39 | Akhter | 2007 | * | * | * | * | ** | * | * | * | 9 |
| 40 | Sugawara | 2009 | * | * | * | * | ** | * | * | * | 9 |
| 41 | Li | 2010 | * | * | * | * | ** | * | * | * | 9 |
| 42 | Kumagai | 2014 | * | * | * | * | ** | * | * | * | 9 |
| **Pooled studies of Japanese cohort studies** | | | |  |  |  |  |  |  |  |  |
| 43 | Mizoue | 2008 | * | * | * | * | ** | * | * |  | 8 |
| 44 | Kashino | 2018 | * | * | * | * | ** | * | * |  | 8 |
| 45 | Islam | 2019 | * | * | * | * | ** | * | * |  | 8 |
| **Jiashan Study** | |  |  |  |  |  |  |  |  |  |  |
| 46 | Chen | 2005 | * | * | * | * | ** | * | * | * | 9 |
| **CKB Study** |  |  |  |  |  |  |  |  |  |  |  |
| 47 | Li | 2019 | * | * | * | * | ** | * | * | * | 9 |
| 48 | Im | 2021 | * | * | * | * | ** | * | * | * | 9 |
| 49 | Chan | 2021 | * | * | * | * | ** | * | * | * | 9 |
| 50 | Kakkoura | 2022 | * | * | * | * | ** | * | * | * | 9 |
| **SWHS Study** | |  |  |  |  |  |  |  |  |  |  |
| 51 | Shin | 2006 | * | * | * | * | ** | * | * | * | 9 |
| 52 | Yang | 2007 | * | * | * | * | ** | * | * | * | 9 |
| 53 | Shrubsole | 2009 | * | * | * | * | ** | * | * | * | 9 |
| 54 | Murff | 2009 | * | * | * | * | ** | * | * | * | 9 |
| 55 | Yang | 2009 | * | * | * | * | ** | * | * | * | 9 |
| 56 | Lee | 2009 | * | * | * | * | ** | * | * | * | 9 |
| 57 | Li | 2011 | * | * | * | * | ** | * | * | * | 9 |
| 58 | Nechuta | 2012 | * | * | * | * | ** | * | * | * | 9 |
| 59 | Pradhan | 2023 | * | * | * | * | ** | * | * | * | 9 |
| **SMHS Study** | |  |  |  |  |  |  |  |  |  |  |
| 60 | Yang | 2011 | * | * | * | * | ** | * | * |  | 8 |
| 61 | Vogtmann | 2013 | * | * | * | * | ** | * | * |  | 8 |
| 62 | Nguyen | 2020 | * | * | * | * | ** | * | * |  | 8 |
| 63 | Nguyen | 2021 | * | * | * | * | ** | * | * |  | 8 |
| **Pooled study of Japanese and Chinese cohort studies** | | | |  |  |  |  |  |  |  |  |
| 64 | Khankari | 2020 | * | * | * | * | ** | * | * |  | 8 |
| **NHIS Study** |  |  |  |  |  |  |  |  |  |  |  |
| 65 | Kim | 2011 | * | * | * | * | ** | * | * |  | 8 |
| 66 | Choi | 2017 | * | * | * | * | ** | * | * |  | 8 |
| 67 | Yoo | 2021 | * | * | * | * | ** | * | * |  | 8 |
| 68 | Bui | 2022 | * | * | * | * | ** | * | * |  | 8 |
| 69 | Yoo | 2022 | * | * | * | * | ** | * | * |  | 8 |
| **HEXA Study** |  |  |  |  |  |  |  |  |  |  |  |
| 70 | Lee | 2021 | * | * | * | * | ** | * | * |  | 8 |
| 71 | Na | 2022 | * | * | * | * | ** | * | * |  | 8 |
| **CSEC Study** |  |  |  |  |  |  |  |  |  |  |  |
| 72 | Wie | 2014 | * | * | * | * | ** | * | * |  | 8 |
| **KMCC Study** | |  |  |  |  |  |  |  |  |  |  |
| 73 | Cho | 2015 | * | * | * | * | ** | * | * | * | 9 |
| **SCHS Study** | |  |  |  |  |  |  |  |  |  |  |
| 74 | Tsong | 2007 | * | * | * | * | ** | * | * | * | 9 |
| 75 | Sun | 2007 | * | * | * | * | ** | * | * | * | 9 |
| 76 | Butler | 2008 | * | * | * | * | ** | * | * | * | 9 |
| 77 | Butler | 2009 | * | * | * | * | ** | * | * | * | 9 |
| 78 | Peterson | 2010 | * | * | * | * | ** | * | * | * | 9 |
| 79 | Yu | 2022 | * | * | * | * | ** | * | * | * | 9 |
| 80 | Yu | 2022 | * | * | * | * | ** | * | * | * | 9 |
| 81 | Yu | 2023 | * | * | * | * | ** | * | * | * | 9 |
| **Taiwanese Study** | |  |  |  |  |  |  |  |  |  |  |
| 82 | Yeh | 2006 | * | * | * | * | ** | * | * | * | 9 |

Studies with total scores ≥7 stars are considered high-quality, studies with total scores of 5–6 stars are considered medium-quality, and studies with total scores <5 stars are considered low-quality.

JPHC: Japan Public Health Center; JACC: the Japan Collaborative Cohort Study; Takayama: the Takayama Cohort Study; Miyagi: the Miyagi Cohort Study; Ohsaki: the Ohsaki National Health Insurance Cohort Study; Jiashan study: population-based cohort study in Jiashan County; CKB: the China Kadoorie Biobank; SWHS: the Shanghai Women’s Health Study; SMHS: the Shanghai Men’s Health Study; NHIS: the National Health Insurance System; HEXA: the Health Examinees Study; CSEC: the Cancer Screening Examination Cohort; KMCC: The Korean Multi-Center Cancer Cohort; SCHS: the Singapore Chinese Health Study

**Supplementary Table S5.** Summary of included cohort studies

| **Country** | **Study** | **Design** | **Age range, years** | **Dietary assessment method** |
| --- | --- | --- | --- | --- |
| Japan | JPHC | Prospective cohort study | 40-74 | Food frequency questionnaire |
|  | JACC | Prospective cohort study | 40-79 | Food frequency questionnaire |
|  | Takayama | Prospective cohort study | ≥35 | Food frequency questionnaire |
|  | Miyagi | Prospective cohort study | 40-64 | Food frequency questionnaire |
|  | Ohsaki | Prospective cohort study | 40-79 | Food frequency questionnaire |
|  | 3-pref Miyagi | Prospective cohort study | ≥40 | Food frequency questionnaire |
|  | 3-pref Aichi | Prospective cohort study | ≥40 | Food frequency questionnaire |
|  | 3-pref Ohsaki | Prospective cohort study | 40- 97 | Food frequency questionnaire |
| China | Jiashan | Prospective cohort study | ≥30 | A self-administered questionnaire |
|  | CKB | Prospective cohort study | 30-79 | Food frequency questionnaire |
|  | SWHS | Prospective cohort study | 40-70 | Food frequency questionnaire |
|  | SMHS | Prospective cohort study | 40-74 | Food frequency questionnaire |
| Korea | HEXA | Prospective cohort study | 40-69 | Food frequency questionnaire |
|  | NHIS | Retrospective cohort study | >20 | A self-administered questionnaire |
|  | CSEC | Prospective cohort study | - | 3-day Dietary record |
|  | KMCC | Prospective cohort study | >20 | Direct interview* |
| Singapore | SCHS | Prospective cohort study | 45-74 | Food frequency questionnaire |
| Taiwan | - | Prospective cohort study | 30-65 | Food frequency questionnaire |

JPHC: Japan Public Health Center; JACC: the Japan Collaborative Cohort Study; Takayama: the Takayama Cohort Study; Miyagi: the Miyagi Cohort Study; Ohsaki: the Ohsaki National Health Insurance Cohort Study; 3-pref Miyagi: the Three Prefecture Study–Miyagi portion; 3-pref Aichi: the Three Prefecture Study–Aichi portion; 3-pref Osaka: the Three Prefecture Study–Osaka portion; Jiashan study: population-based cohort study in Jiashan County; CKB: the China Kadoorie Biobank; SWHS: the Shanghai Women’s Health Study; SMHS: the Shanghai Men’s Health Study; NHIS: the National Health Insurance System; HEXA: the Health Examinees Study; CSEC: the Cancer Screening Examination Cohort; KMCC: The Korean Multi-Center Cancer Cohort; SCHS: the Singapore Chinese Health Study

*Data on alcohol consumption was collected by direct interview at baseline

**Supplementary Table S6.** Pooled results for food groups, dietary patterns, and colorectal cancer risk

| **Exposure** | **Cancer site** | **No. Study** | **RR (95% CI)** | **I^2^ (%)** | **P for  heterogeneity** | **Subgroup analysis** |
| --- | --- | --- | --- | --- | --- | --- |
| Total meat |  |  |  |  |  |  |
|  | Colorectum | 6 | **1.18 (1.03, 1.34)** | 51.7 | 0.066 | ★ |
|  | Colon | 4 | **1.30 (1.08, 1.58)** | 0.0 | 0.777 |  |
|  | Rectum | 4 | 0.82 (0.63, 1.07) | 0.0 | 0.897 |  |
| Red meat |  |  |  |  |  |  |
|  | Colorectum | 10 | 1.07 (0.96, 1.20) | 26.2 | 0.202 |  |
|  | Colon | 7 | 1.06 (0.91, 1.25) | 0.0 | 0.835 |  |
|  | Rectum | 7 | 1.19 (0.91, 1.54) | 35.8 | 0.155 |  |
| Processed meat |  |  |  |  |  |  |
|  | Colorectum | 5 | **1.18 (1.06, 1.32)** | 0.0 | 0.905 |  |
|  | Colon | 4 | **1.24 (1.03, 1.50)** | 0.0 | 0.408 |  |
|  | Rectum | 4 | 1.14 (0.90, 1.44) | 0.0 | 0.490 |  |
| White meat |  |  |  |  |  |  |
|  | Colorectum | 3 | 1.08 (0.89, 1.32) | 0.0 | 0.709 |  |
|  | Colon | 3 | 1.00 (0.76, 1.30) | 0.0 | 0.421 |  |
|  | Rectum | 3 | **1.40 (1.00, 1.96)** | 0.0 | 0.945 |  |
| Fruits and vegetables |  |  |  |  |  |  |
|  | Colorectum | 3 | 0.94 (0.80, 1.11) | 41.1 | 0.183 |  |
|  | Colon | 2 | 0.84 (0.56, 1.27) | 30.7 | 0.230 |  |
|  | Rectum | 2 | 0.88 (0.58, 1.32) | 0.0 | 0.387 |  |
| Fruits |  |  |  |  |  |  |
|  | Colorectum | 6 | 0.95 (0.83, 1.08) | 47.7 | 0.089 | ★ |
|  | Colon | 4 | 1.00 (0.81, 1.24) | 16.2 | 0.310 |  |
|  | Rectum | 4 | 0.97 (0.63, 1.49) | 58.2 | 0.066 |  |
| Vegetables |  |  |  |  |  |  |
|  | Colorectum | 6 | 1.00 (0.91, 1.09) | 0.0 | 0.823 |  |
|  | Colon | 4 | 1.06 (0.87, 1.29) | 0.0 | 0.812 |  |
|  | Rectum | 4 | 0.90 (0.66, 1.23) | 14.3 | 0.321 |  |
| Soy food |  |  |  |  |  |  |
|  | Colorectum | 4 | 0.89 (0.75, 1.05) | 36.4 | 0.194 | ★ |
|  | Colon | 5 | 0.86 (0.67, 1.10) | 48.8 | 0.099 |  |
|  | Rectum | 3 | 0.84 (0.52, 1.35) | 60.3 | 0.080 |  |
| Soy isoflavone |  |  |  |  |  |  |
|  | Colorectum | 2 | 0.94 (0.81, 1.09) | 21.9 | 0.258 |  |
|  | Colon | 3 | 1.02 (0.76, 1.37) | 48.8 | 0.142 |  |
|  | Rectum | . |  |  |  |  |
| Fish |  |  |  |  |  |  |
|  | Colorectum | 4 | 1.14 (0.98, 1.32) | 0.0 | 0.778 |  |
|  | Colon | 6 | 1.06 (0.84, 1.33) | 0.0 | 0.559 |  |
|  | Rectum | 6 | 1.01 (0.76, 1.34) | 0.0 | 0.465 |  |
| Alcohol |  |  |  |  |  |  |
|  | Colorectum | 8 | **1.64 (1.20, 2.24)** | 85.4 | 0.000 | ★ |
|  | Colon | 2 | **2.41 (1.18, 4.93)** | 87.5 | 0.005 |  |
|  | Rectum | 2 | **2.22 (1.40, 3.50)** | 0.0 | 0.785 |  |
| Green Tea |  |  |  |  |  |  |
|  | Colorectum | 9 | 0.97 (0.88, 1.07) | 0.0 | 0.507 |  |
|  | Colon | 8 | 0.95 (0.81, 1.12) | 0.0 | 0.843 |  |
|  | Rectum | 8 | 0.99 (0.80, 1.22) | 3.0 | 0.407 |  |
| Coffee |  |  |  |  |  |  |
|  | Colorectum | 4 | 0.97 (0.85, 1.11) | 41.4 | 0.163 | ★ |
|  | Colon | 7 | 0.89 (0.77, 1.03) | 45.3 | 0.089 |  |
|  | Rectum | 5 | 1.08 (0.96, 1.23) | 0.0 | 0.946 |  |
| Calcium |  |  |  |  |  |  |
|  | Colorectum | 5 | **0.93 (0.86, 1.00)** | 0.0 | 0.503 |  |
|  | Colon | 3 | **0.90 (0.81, 1.00)** | 0.0 | 0.402 |  |
|  | Rectum | 3 | 1.00 (0.87, 1.14) | 0.0 | 0.577 |  |
| Fiber |  |  |  |  |  |  |
|  | Colorectum | 5 | 1.00 (0.90, 1.11) | 0.0 | 0.450 | ★ |
|  | Colon | 3 | 0.90 (0.56, 1.43) | 70.3 | 0.034 |  |
|  | Rectum | 3 | 1.01 (0.75, 1.37) | 0.0 | 0.930 |  |
| Healthy Pattern |  |  |  |  |  |  |
|  | Colorectum | 4 | **0.90 (0.80, 1.01)** | 31.4 | 0.224 | ★ |
|  | Colon | 3 | **0.85 (0.74, 0.97)** | 0.0 | 0.885 |  |
|  | Rectum | 3 | 0.90 (0.53, 1.52) | 84.4 | 0.002 |  |
| Traditional Pattern |  |  |  |  |  |  |
|  | Colorectum | 3 | 1.00 (0.89, 1.13) | 0.0 | 0.921 |  |
|  | Colon | 3 | 1.04 (0.90, 1.21) | 0.0 | 0.822 |  |
|  | Rectum | 3 | 0.90 (0.72, 1.12) | 0.0 | 0.514 |  |
| Unhealthy pattern |  |  |  |  |  |  |
|  | Colorectum | 4 | 0.99 (0.89, 1.11) | 16.5 | 0.309 | ★ |
|  | Colon | 3 | 1.01 (0.81, 1.26) | 56.4 | 0.101 |  |
|  | Rectum | 3 | 0.97 (0.76, 1.25) | 19.6 | 0.230 |  |

RR: relative risk; CI: confidence interval


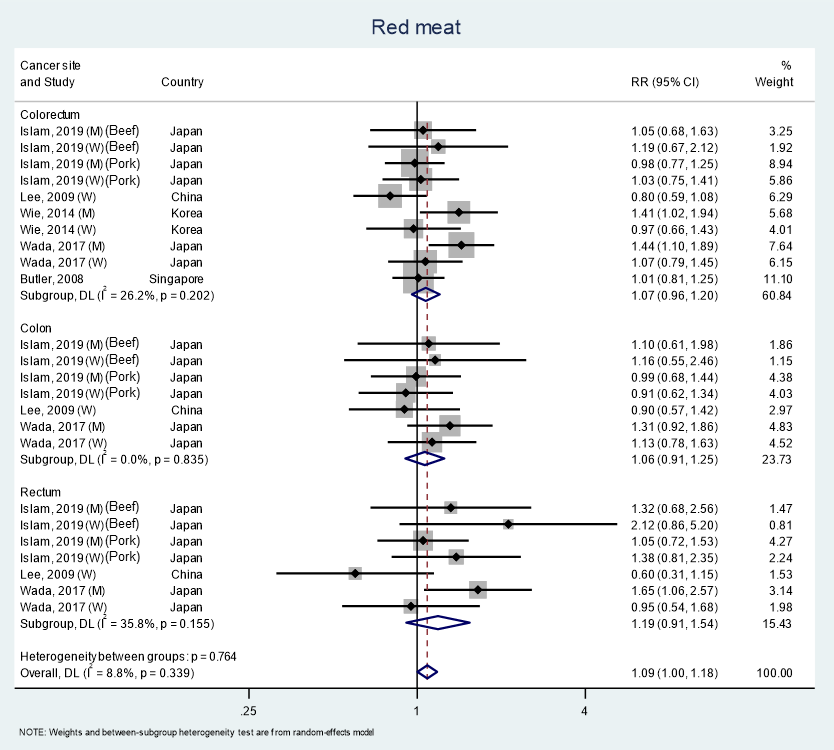

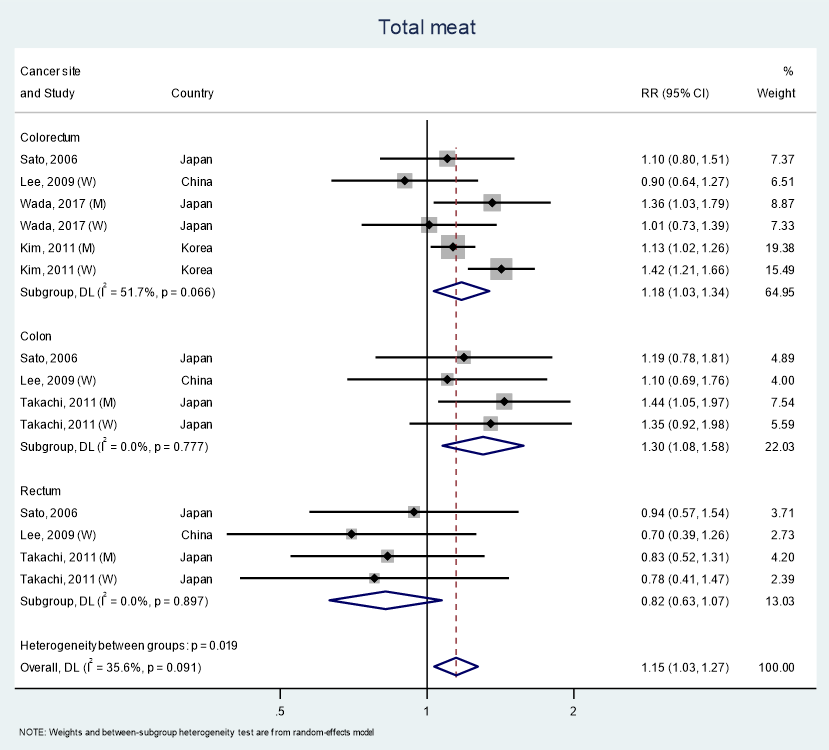


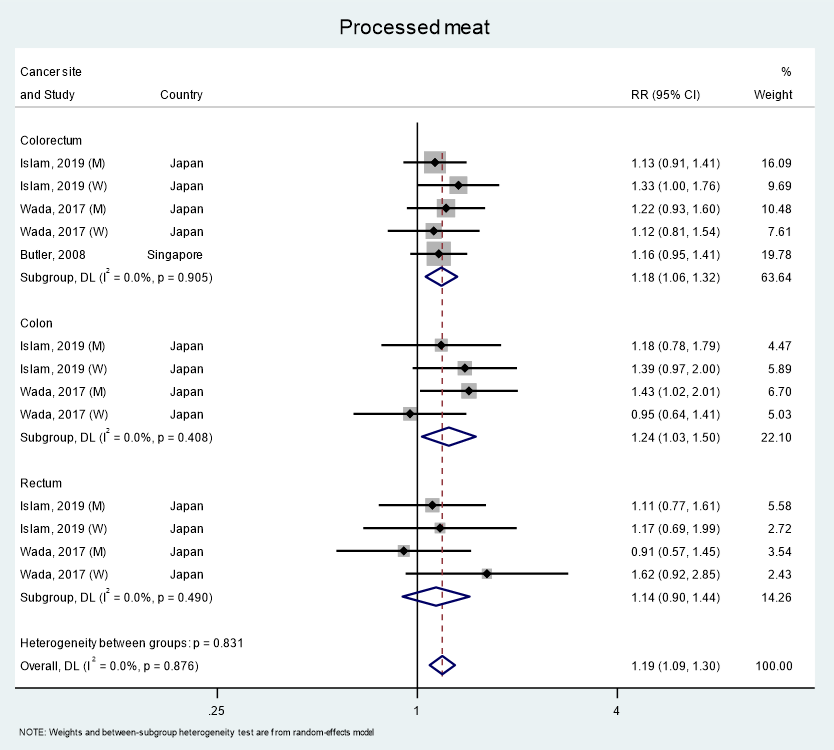
(A) (B)

(C) (D)

**Supplementary** **Figure S1.** Forest plots: associations between food groups, dietary patterns, and the incidence of colorectal, colon, and rectal cancer. (A) Total meat (B) red meat (C) processed meat (D) white meat

(E) (F)

 (G) (H)

**(continued) Supplementary** **Figure S1.** Forest plots: associations between food groups, dietary patterns, and the incidence of colorectal, colon, and rectal cancer. (E) Total fruits and vegetables, (F) fruits, (G) vegetables, and (H) soy foods

(I) (J)


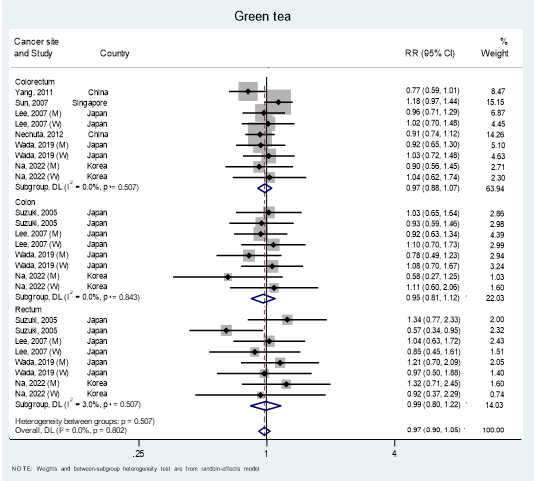


(K) (L)

**(continued) Supplementary** **Figure S1.** Forest plots: associations between food groups, dietary patterns, and the incidence of colorectal, colon, and rectal cancer. (I) Soy isoflavone (J) fish (K) alcohol (L) green tea

 (M) (N)

(O) (P)

**(continued) Supplementary** **Figure S1.** Forest plots: associations between food groups, dietary patterns, and the incidence of colorectal, colon, and rectal cancer. (M) Coffee (N) calcium (O) fiber (P) healthy pattern

(Q) (R)

**(continued) Supplementary** **Figure S1.** Forest plots: associations between food groups, dietary patterns, and the incidence of colorectal, colon, and rectal cancer. (Q) Traditional pattern (R) unhealthy pattern

(A) (B)

(C) (D)

(E) (F)

**Supplementary** **Figure S2.** Publication bias: associations between food groups, dietary patterns and the incidence of colorectal, colon, and rectal cancer. (A) Total meat, (Egger's test: *P* = 0.161, Begg's test: *P* = 0.025); (B) red meat, (Egger's test: *P* = 0.536, Begg's test: *P* = 0.399); (C) processed meat, (Egger's test: *P* = 0.810, Begg's test: *P* = 0.807); (D) fruit, (Egger's test: *P* = 0.846, Begg's test: *P* = 0.870); (E) vegetables, (Egger's test: *P* = 0.761, Begg's test: *P* = 0.547); (F) soy food, (Egger's test: *P* = 0.599, Begg's test: *P* = 0.784)


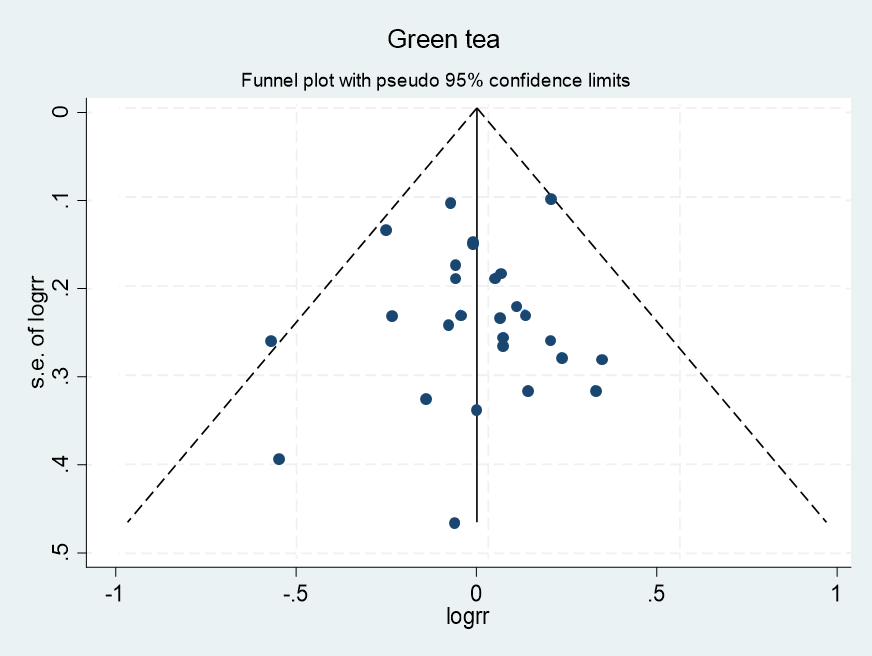
****(G) (H)

(I) (J)

(K) (L)

**(continued) Supplementary** **Figure S2.** Publication bias: associations between food groups, dietary patterns and he incidence of colorectal, colon, and rectal cancer. (G) Fish, (Egger's test: *P* = 0.221, Begg's test: *P* = 0.126); (H) alcohol, (Egger's test: *P* = 0.035, Begg's test: *P* = 0.217); (I) green tea, (Egger's test: *P* = 0.691, Begg's test: *P* = 0.640); (J) coffee, (Egger's test: *P* = 0.388, Begg's test: *P* = 0.528); (K) calcium, (Egger's test: *P* = 0.064, Begg's test: *P* = 0.392); (L) fiber, (Egger's test: *P* = 0.529, Begg's test: *P* = 0.484)

(M) (N)

**Supplementary** **Figure S2.** Publication bias: associations between food groups, dietary patterns, and the incidence of colorectal, colon, and rectal cancer. (M) Healthy pattern, (Egger's test: *P* = 0.973, Begg's test: *P* = 0.929); (N) unhealthy pattern, (Egger's test: *P* = 0.510, Begg's test: *P* = 0.421)

**References**

1. Otani T, Iwasaki M, Yamamoto S, Sobue T, Hanaoka T, Inoue M, et al. Alcohol consumption, smoking, and subsequent risk of colorectal cancer in middle-aged and elderly Japanese men and women: Japan Public Health Center-based prospective study. Cancer Epidemiol Biomarkers Prev. 2003;12(12):1492-500

2. Kobayashi M, Tsubono Y, Otani T, Hanaoka T, Sobue T, Tsugane S. Fish, long-chain n-3 polyunsaturated fatty acids, and risk of colorectal cancer in middle-aged Japanese: the JPHC study. Nutr Cancer. 2004;49(1):32-40.<https://doi.org/10.1207/s15327914nc4901_5>

3. Tsubono Y, Otani T, Kobayashi M, Yamamoto S, Sobue T, Tsugane S. No association between fruit or vegetable consumption and the risk of colorectal cancer in Japan. Br J Cancer. 2005;92(9):1782-4.<https://doi.org/10.1038/sj.bjc.6602566>

4. Kim MK, Sasaki S, Otani T, Tsugane S. Dietary patterns and subsequent colorectal cancer risk by subsite: a prospective cohort study. Int J Cancer. 2005;115(5):790-8.<https://doi.org/10.1002/ijc.20943>

5. Otani T, Iwasaki M, Ishihara J, Sasazuki S, Inoue M, Tsugane S. Dietary fiber intake and subsequent risk of colorectal cancer: the Japan Public Health Center-based prospective study. Int J Cancer. 2006;119(6):1475-80.<https://doi.org/10.1002/ijc.22007>

6. Lee KJ, Inoue M, Otani T, Iwasaki M, Sasazuki S, Tsugane S. Coffee consumption and risk of colorectal cancer in a population-based prospective cohort of Japanese men and women. Int J Cancer. 2007;121(6):1312-8.<https://doi.org/10.1002/ijc.22778>

7. Ishihara J, Otani T, Inoue M, Iwasaki M, Sasazuki S, Tsugane S. Low intake of vitamin B-6 is associated with increased risk of colorectal cancer in Japanese men. J Nutr. 2007;137(7):1808-14.<https://doi.org/10.1093/jn/137.7.1808>

8. Akhter M, Inoue M, Kurahashi N, Iwasaki M, Sasazuki S, Tsugane S. Dietary soy and isoflavone intake and risk of colorectal cancer in the Japan public health center-based prospective study. Cancer Epidemiol Biomarkers Prev. 2008;17(8):2128-35.<https://doi.org/10.1158/1055-9965.Epi-08-0182>

9. Ishihara J, Inoue M, Iwasaki M, Sasazuki S, Tsugane S. Dietary calcium, vitamin D, and the risk of colorectal cancer. Am J Clin Nutr. 2008;88(6):1576-83.<https://doi.org/10.3945/ajcn.2008.26195>

10. Ma E, Sasazuki S, Inoue M, Iwasaki M, Sawada N, Takachi R, et al. High dietary intake of magnesium may decrease risk of colorectal cancer in Japanese men. J Nutr. 2010;140(4):779-85.<https://doi.org/10.3945/jn.109.117747>

11. Takachi R, Inoue M, Shimazu T, Sasazuki S, Ishihara J, Sawada N, et al. Consumption of sodium and salted foods in relation to cancer and cardiovascular disease: the Japan Public Health Center-based Prospective Study. Am J Clin Nutr. 2010;91(2):456-64.<https://doi.org/10.3945/ajcn.2009.28587>

12. Takachi R, Tsubono Y, Baba K, Inoue M, Sasazuki S, Iwasaki M, et al. Red meat intake may increase the risk of colon cancer in Japanese, a population with relatively low red meat consumption. Asia Pacific journal of clinical nutrition. 2011;20(4):603-12

13. Sasazuki S, Inoue M, Iwasaki M, Sawada N, Shimazu T, Yamaji T, et al. Intake of n-3 and n-6 polyunsaturated fatty acids and development of colorectal cancer by subsite: Japan Public Health Center-based prospective study. Int J Cancer. 2011;129(7):1718-29.<https://doi.org/10.1002/ijc.25802>

14. Hara A, Sasazuki S, Inoue M, Iwasaki M, Shimazu T, Sawada N, et al. Zinc and heme iron intakes and risk of colorectal cancer: a population-based prospective cohort study in Japan. Am J Clin Nutr. 2012;96(4):864-73.<https://doi.org/10.3945/ajcn.112.041202>

15. Abe SK, Inoue M, Sawada N, Iwasaki M, Ishihara J, Sasazuki S, et al. Rice, bread, noodle and cereal intake and colorectal cancer in Japanese men and women: the Japan Public Health Center-based prospective Study (JPHC Study). Br J Cancer. 2014;110(5):1316-21.<https://doi.org/10.1038/bjc.2013.799>

16. Abe SK, Inoue M, Sawada N, Ishihara J, Iwasaki M, Yamaji T, et al. Glycemic index and glycemic load and risk of colorectal cancer: a population-based cohort study (JPHC Study). Cancer Causes & Control. 2016;27(4):583-93.<https://doi.org/10.1007/s10552-016-0733-6>

17. Shin S, Saito E, Sawada N, Ishihara J, Takachi R, Nanri A, et al. Dietary patterns and colorectal cancer risk in middle-aged adults: A large population-based prospective cohort study. Clin Nutr. 2018;37(3):1019-26.<https://doi.org/10.1016/j.clnu.2017.04.015>

18. Mori N, Sawada N, Shimazu T, Yamaji T, Goto A, Takachi R, et al. Cruciferous vegetable intake and colorectal cancer risk: Japan public health center-based prospective study. Eur J Cancer Prev. 2019;28(5):420-7.<https://doi.org/10.1097/cej.0000000000000491>

19. Leung CY, Abe SK, Sawada N, Ishihara J, Takachi R, Yamaji T, et al. Sugary Drink Consumption and Subsequent Colorectal Cancer Risk: The Japan Public Health Center-Based Prospective Cohort Study. Cancer Epidemiol Biomarkers Prev. 2021;30(4):782-8.<https://doi.org/10.1158/1055-9965.Epi-20-1364>

20. Cai H, Sobue T, Kitamura T, Ishihara J, Nanri A, Mizoue T, et al. Low-carbohydrate diet and risk of cancer incidence: The Japan Public Health Center-based prospective study. Cancer Sci. 2022;113(2):744-55.<https://doi.org/10.1111/cas.15215>

21. Kanehara R, Katagiri R, Goto A, Yamaji T, Sawada N, Iwasaki M, et al. Sugar intake and colorectal cancer risk: A prospective Japanese cohort study. Cancer Sci. 2023;114(6):2584-95.<https://doi.org/10.1111/cas.15766>

22. Hori M, Sawada N, Kito K, Yamaji T, Iwasaki M, Inoue M, et al. Vegetable and fruit intake and colorectal cancer risk by smoking status in adults: The Japan Public Health Center-based Prospective Study. Eur J Clin Nutr. 2023;77(2):255-63.<https://doi.org/10.1038/s41430-022-01214-2>

23. Wakai K, Kojima M, Tamakoshi K, Watanabe Y, Hayakawa N, Suzuki K, et al. Alcohol consumption and colorectal cancer risk: Findings from the JACC Study. Journal of Epidemiology. 2005;15(SUPPL. 2):S173-S9.<https://doi.org/10.2188/jea.15.S173>

24. Wakai K, Date C, Fukui M, Tamakoshi K, Watanabe Y, Hayakawa N, et al. Dietary fiber and risk of colorectal cancer in the Japan collaborative cohort study. Cancer Epidemiol Biomarkers Prev. 2007;16(4):668-75.<https://doi.org/10.1158/1055-9965.Epi-06-0664>

25. Aoyama N, Kawado M, Yamada H, Hashimoto S, Suzuki K, Wakai K, et al. Low intake of vegetables and fruits and risk of colorectal cancer: the Japan Collaborative Cohort Study. J Epidemiol. 2014;24(5):353-60.<https://doi.org/10.2188/jea.je20130195>

26. Yamada H, Kawado M, Aoyama N, Hashimoto S, Suzuki K, Wakai K, et al. Coffee consumption and risk of colorectal cancer: the Japan Collaborative Cohort Study. J Epidemiol. 2014;24(5):370-8.<https://doi.org/10.2188/jea.je20130168>

27. Kato A, Okada C, Eshak ES, Iso H, Tamakoshi A. Association between dietary intake of n-3 polyunsaturated fatty acids and risk of colorectal cancer in the Japanese population: The Japan Collaborative Cohort Study. Cancer Med. 2023;12(4):4690-700.<https://doi.org/10.1002/cam4.5098>

28. Shimizu N, Nagata C, Shimizu H, Kametani M, Takeyama N, Ohnuma T, et al. Height, weight, and alcohol consumption in relation to the risk of colorectal cancer in Japan: a prospective study. Br J Cancer. 2003;88(7):1038-43.<https://doi.org/10.1038/sj.bjc.6600845>

29. Oba S, Shimizu N, Nagata C, Shimizu H, Kametani M, Takeyama N, et al. The relationship between the consumption of meat, fat, and coffee and the risk of colon cancer: a prospective study in Japan. Cancer Lett. 2006;244(2):260-7.<https://doi.org/10.1016/j.canlet.2005.12.037>

30. Oba S, Nagata C, Shimizu N, Shimizu H, Kametani M, Takeyama N, et al. Soy product consumption and the risk of colon cancer: a prospective study in Takayama, Japan. Nutr Cancer. 2007;57(2):151-7.<https://doi.org/10.1080/01635580701274475>

31. Wada K, Oba S, Tsuji M, Tamura T, Konishi K, Goto Y, et al. Meat consumption and colorectal cancer risk in Japan: The Takayama study. Cancer Sci. 2017;108(5):1065-70.<https://doi.org/10.1111/cas.13217>

32. Wada K, Oba S, Tsuji M, Goto Y, Mizuta F, Koda S, et al. Green tea intake and colorectal cancer risk in Japan: the Takayama study. Jpn J Clin Oncol. 2019;49(6):515-20.<https://doi.org/10.1093/jjco/hyz030>

33. Wada K, Nakashima Y, Yamakawa M, Hori A, Seishima M, Tanabashi S, et al. Dietary advanced glycation end products and cancer risk in Japan: From the Takayama study. Cancer Science. 2022;113(8):2839-48.<https://doi.org/10.1111/cas.15455>

34. Nakaya N, Tsubono Y, Kuriyama S, Hozawa A, Shimazu T, Kurashima K, et al. Alcohol consumption and the risk of cancer in Japanese men: the Miyagi cohort study. European Journal of Cancer Prevention. 2005;14(2):169-74.<https://doi.org/10.1097/00008469-200504000-00013>

35. Suzuki Y, Tsubono Y, Nakaya N, Koizumi Y, Suzuki Y, Shibuya D, et al. Green tea and the risk of colorectal cancer: pooled analysis of two prospective studies in Japan. J Epidemiol. 2005;15(4):118-24.<https://doi.org/10.2188/jea.15.118>

36. Sato Y, Tsubono Y, Nakaya N, Ogawa K, Kurashima K, Kuriyama S, et al. Fruit and vegetable consumption and risk of colorectal cancer in Japan: The Miyagi Cohort Study. Public Health Nutr. 2005;8(3):309-14.<https://doi.org/10.1079/phn2004681>

37. Sato Y, Nakaya N, Kuriyama S, Nishino Y, Tsubono Y, Tsuji I. Meat consumption and risk of colorectal cancer in Japan: the Miyagi Cohort Study. Eur J Cancer Prev. 2006;15(3):211-8.<https://doi.org/10.1097/01.cej.0000197455.87356.05>

38. Naganuma T, Kuriyama S, Akhter M, Kakizaki M, Nakaya N, Matsuda-Ohmori K, et al. Coffee consumption and the risk of colorectal cancer: a prospective cohort study in Japan. Int J Cancer. 2007;120(7):1542-7.<https://doi.org/10.1002/ijc.22505>

39. Akhter M, Kuriyama S, Nakaya N, Shimazu T, Ohmori K, Nishino Y, et al. Alcohol consumption is associated with an increased risk of distal colon and rectal cancer in Japanese men: the Miyagi Cohort Study. Eur J Cancer. 2007;43(2):383-90.<https://doi.org/10.1016/j.ejca.2006.09.020>

40. Sugawara Y, Kuriyama S, Kakizaki M, Nagai M, Ohmori-Matsuda K, Sone T, et al. Fish consumption and the risk of colorectal cancer: the Ohsaki Cohort Study. Br J Cancer. 2009;101(5):849-54.<https://doi.org/10.1038/sj.bjc.6605217>

41. Li WQ, Kuriyama S, Li Q, Nagai M, Hozawa A, Nishino Y, et al. Citrus consumption and cancer incidence: The Ohsaki cohort study. International Journal of Cancer. 2010;127(8):1913-22.<https://doi.org/10.1002/ijc.25203>

42. Kumagai Y, Chou WT, Tomata Y, Sugawara Y, Kakizaki M, Nishino Y, et al. Dietary patterns and colorectal cancer risk in Japan: the Ohsaki Cohort Study. Cancer Causes Control. 2014;25(6):727-36.<https://doi.org/10.1007/s10552-014-0375-5>

43. Mizoue T, Inoue M, Wakai K, Nagata C, Shimazu T, Tsuji I, et al. Alcohol drinking and colorectal cancer in Japanese: a pooled analysis of results from five cohort studies. American journal of epidemiology. 2008;167(12):1397-406.<https://doi.org/10.1093/aje/kwn073>

44. Kashino I, Akter S, Mizoue T, Sawada N, Kotemori A, Matsuo K, et al. Coffee drinking and colorectal cancer and its subsites: A pooled analysis of 8 cohort studies in Japan. Int J Cancer. 2018;143(2):307-16.<https://doi.org/10.1002/ijc.31320>

45. Islam Z, Akter S, Kashino I, Mizoue T, Sawada N, Mori N, et al. Meat subtypes and colorectal cancer risk: A pooled analysis of 6 cohort studies in Japan. Cancer Science. 2019;110(11):3603-14.<https://doi.org/10.1111/cas.14188>

46. Chen K, Jiang Q, Ma X, Li Q, Yao K, Yu W, et al. Alcohol drinking and colorectal cancer: a population-based prospective cohort study in China. Eur J Epidemiol. 2005;20(2):149-54.<https://doi.org/10.1007/s10654-004-2953-4>

47. Li X, Yu C, Guo Y, Bian Z, Shen Z, Yang L, et al. Association between tea consumption and risk of cancer: a prospective cohort study of 0.5 million Chinese adults. European Journal of Epidemiology. 2019;34(8):753-63.<https://doi.org/10.1007/s10654-019-00530-5>

48. Im PK, Millwood IY, Kartsonaki C, Chen Y, Guo Y, Du H, et al. Alcohol drinking and risks of total and site-specific cancers in China: A 10-year prospective study of 0.5 million adults. Int J Cancer. 2021;149(3):522-34.<https://doi.org/10.1002/ijc.33538>

49. Chan WC, Millwood IY, Kartsonaki C, Du H, Guo Y, Chen Y, et al. Spicy food consumption and risk of gastrointestinal-tract cancers: findings from the China Kadoorie Biobank. Int J Epidemiol. 2021;50(1):199-211.<https://doi.org/10.1093/ije/dyaa275>

50. Kakkoura MG, Du H, Guo Y, Yu C, Yang L, Pei P, et al. Dairy consumption and risks of total and site-specific cancers in Chinese adults: an 11-year prospective study of 0.5 million people. BMC Med. 2022;20(1):134.<https://doi.org/10.1186/s12916-022-02330-3>

51. Shin A, Li H, Shu XO, Yang G, Gao YT, Zheng W. Dietary intake of calcium, fiber and other micronutrients in relation to colorectal cancer risk: Results from the Shanghai Women's Health Study. International Journal of Cancer. 2006;119(12):2938-42.<https://doi.org/10.1002/ijc.22196>

52. Yang G, Shu XO, Li H, Chow WH, Ji BT, Zhang X, et al. Prospective cohort study of green tea consumption and colorectal cancer risk in women. Cancer Epidemiol Biomarkers Prev. 2007;16(6):1219-23.<https://doi.org/10.1158/1055-9965.Epi-07-0097>

53. Shrubsole MJ, Yang G, Gao YT, Chow WH, Shu XO, Cai Q, et al. Dietary B vitamin and methionine intakes and plasma folate are not associated with colorectal cancer risk in Chinese women. Cancer Epidemiol Biomarkers Prev. 2009;18(3):1003-6.<https://doi.org/10.1158/1055-9965.Epi-08-1200>

54. Murff HJ, Shu XO, Li H, Dai Q, Kallianpur A, Yang G, et al. A prospective study of dietary polyunsaturated fatty acids and colorectal cancer risk in Chinese women. Cancer Epidemiol Biomarkers Prev. 2009;18(8):2283-91.<https://doi.org/10.1158/1055-9965.Epi-08-1196>

55. Yang G, Shu XO, Li H, Chow WH, Cai H, Zhang X, et al. Prospective cohort study of soy food intake and colorectal cancer risk in women. Am J Clin Nutr. 2009;89(2):577-83.<https://doi.org/10.3945/ajcn.2008.26742>

56. Lee S-A, Shu XO, Yang G, Li H, Gao Y-T, Zheng W. Animal Origin Foods and Colorectal Cancer Risk: A Report From the Shanghai Women's Health Study. Nutrition and Cancer. 2009;61(2):194-205.<https://doi.org/10.1080/01635580802419780>

57. Li HL, Yang G, Shu XO, Xiang YB, Chow WH, Ji BT, et al. Dietary glycemic load and risk of colorectal cancer in Chinese women. Am J Clin Nutr. 2011;93(1):101-7.<https://doi.org/10.3945/ajcn.110.003053>

58. Nechuta S, Shu XO, Li HL, Yang G, Ji BT, Xiang YB, et al. Prospective cohort study of tea consumption and risk of digestive system cancers: results from the Shanghai Women's Health Study. Am J Clin Nutr. 2012;96(5):1056-63.<https://doi.org/10.3945/ajcn.111.031419>

59. Pradhan P, Wen W, Cai H, Gao YT, Shu XO, Zheng W. Prospective Cohort Study of Ginseng Consumption in Association with Cancer Risk: Shanghai Women's Health Study. Journal of Nutrition. 2023;153(4):1170-7.<https://doi.org/10.1016/j.tjnut.2023.02.032>

60. Yang G, Zheng W, Xiang Y, Gao J, Li H, Zhang X, et al. Green tea consumption and colorectal cancer risk: A report from the shanghai men's health study. Carcinogenesis. 2011;32(11):1684-8.<https://doi.org/10.1093/carcin/bgr186>

61. Vogtmann E, Xiang YB, Li HL, Levitan EB, Yang G, Waterbor JW, et al. Fruit and vegetable intake and the risk of colorectal cancer: results from the Shanghai Men's Health Study. Cancer Causes Control. 2013;24(11):1935-45.<https://doi.org/10.1007/s10552-013-0268-z>

62. Nguyen S, Li H, Yu D, Gao J, Gao Y, Tran H, et al. Adherence to dietary recommendations and colorectal cancer risk: results from two prospective cohort studies. Int J Epidemiol. 2020;49(1):270-80.<https://doi.org/10.1093/ije/dyz118>

63. Nguyen S, Li H, Yu D, Cai H, Gao J, Gao Y, et al. Dietary fatty acids and colorectal cancer risk in men: A report from the Shanghai Men's Health Study and a meta-analysis. Int J Cancer. 2021;148(1):77-89.<https://doi.org/10.1002/ijc.33196>

64. Khankari NK, Yang JJ, Sawada N, Wen W, Yamaji T, Gao J, et al. Soy Intake and Colorectal Cancer Risk: Results from a Pooled Analysis of Prospective Cohort Studies Conducted in China and Japan. J Nutr. 2020;150(9):2442-50.<https://doi.org/10.1093/jn/nxaa194>

65. Kim J, Park S, Nam BH. The risk of colorectal cancer is associated with the frequency of meat consumption in a population-based cohort in Korea. Asian Pac J Cancer Prev. 2011;12(9):2371-6

66. Choi YJ, Lee DH, Han KD, Kim HS, Yoon H, Shin CM, et al. The relationship between drinking alcohol and esophageal, gastric or colorectal cancer: A nationwide population-based cohort study of South Korea. PLoS One. 2017;12(10):e0185778.<https://doi.org/10.1371/journal.pone.0185778>

67. Yoo JE, Shin DW, Han K, Kim D, Jeong SM, Koo HY, et al. Association of the Frequency and Quantity of Alcohol Consumption With Gastrointestinal Cancer. JAMA Netw Open. 2021;4(8):e2120382.<https://doi.org/10.1001/jamanetworkopen.2021.20382>

68. Bui TT, Han M, Luu NM, Tran TPT, Lim MK, Oh JK. Cancer risk according to alcohol consumption trajectories: A population-based cohort study on 2.8 million Korean men. J Epidemiol. 2022.<https://doi.org/10.2188/jea.JE20220175>

69. Yoo JE, Han K, Shin DW, Kim D, Kim BS, Chun S, et al. Association Between Changes in Alcohol Consumption and Cancer Risk. JAMA Netw Open. 2022;5(8):e2228544.<https://doi.org/10.1001/jamanetworkopen.2022.28544>

70. Lee J, Shin A, Choi JY, Kang D, Lee JK. Adherence to the Recommended Intake of Calcium and Colorectal Cancer Risk in the HEXA Study. Cancer Res Treat. 2021;53(1):140-7.<https://doi.org/10.4143/crt.2020.480>

71. Na H, Lee J, Cho S, Shin WK, Choi JY, Kang D, et al. Consumption of Coffee and Green Tea and the Risk of Colorectal Cancer in Korea: The Health Examinees Study. J Cancer Prev. 2022;27(4):229-38.<https://doi.org/10.15430/jcp.2022.27.4.229>

72. Wie GA, Cho YA, Kang HH, Ryu KA, Yoo MK, Kim YA, et al. Red meat consumption is associated with an increased overall cancer risk: A prospective cohort study in Korea. British Journal of Nutrition. 2014;112(2):238-47.<https://doi.org/10.1017/S0007114514000683>

73. Cho S, Shin A, Park SK, Shin HR, Chang SH, Yoo KY. Alcohol Drinking, Cigarette Smoking and Risk of Colorectal Cancer in the Korean Multi-center Cancer Cohort. J Cancer Prev. 2015;20(2):147-52.<https://doi.org/10.15430/jcp.2015.20.2.147>

74. Tsong WH, Koh WP, Yuan JM, Wang R, Sun CL, Yu MC. Cigarettes and alcohol in relation to colorectal cancer: the Singapore Chinese Health Study. Br J Cancer. 2007;96(5):821-7.<https://doi.org/10.1038/sj.bjc.6603623>

75. Sun CL, Yuan JM, Koh WP, Lee HP, Yu MC. Green tea and black tea consumption in relation to colorectal cancer risk: the Singapore Chinese Health Study. Carcinogenesis. 2007;28(10):2143-8.<https://doi.org/10.1093/carcin/bgm171>

76. Butler LM, Wang R, Koh WP, Yu MC. Prospective study of dietary patterns and colorectal cancer among Singapore Chinese. Br J Cancer. 2008;99(9):1511-6.<https://doi.org/10.1038/sj.bjc.6604678>

77. Butler LM, Wang R, Koh WP, Stern MC, Yuan JM, Yu MC. Marine n-3 and saturated fatty acids in relation to risk of colorectal cancer in Singapore Chinese: a prospective study. Int J Cancer. 2009;124(3):678-86.<https://doi.org/10.1002/ijc.23950>

78. Peterson S, Yuan JM, Koh WP, Sun CL, Wang R, Turesky RJ, et al. Coffee intake and risk of colorectal cancer among Chinese in Singapore: the Singapore Chinese Health Study. Nutr Cancer. 2010;62(1):21-9.<https://doi.org/10.1080/01635580903191528>

79. Yu YC, Paragomi P, Jin A, Wang R, Schoen RE, Koh WP, et al. Dietary Nonstarch Polysaccharide Intake and Risk of Colorectal Cancer: Findings from the Singapore Chinese Health Study. Cancer Res Commun. 2022;2(10):1304-11.<https://doi.org/10.1158/2767-9764.crc-22-0153>

80. Yu YC, Paragomi P, Wang R, Jin A, Schoen RE, Sheng LT, et al. Composite dietary antioxidant index and the risk of colorectal cancer: Findings from the Singapore Chinese Health Study. Int J Cancer. 2022;150(10):1599-608.<https://doi.org/10.1002/ijc.33925>

81. Yu YC, Paragomi P, Jin A, Wang R, Schoen RE, Koh WP, et al. Low-Carbohydrate Diet Score and the Risk of Colorectal Cancer: Findings from the Singapore Chinese Health Study. Cancer Epidemiol Biomarkers Prev. 2023;32(6):802-8.<https://doi.org/10.1158/1055-9965.Epi-22-0683>

82. Yeh CC, You SL, Chen CJ, Sung FC. Peanut consumption and reduced risk of colorectal cancer in women: a prospective study in Taiwan. World J Gastroenterol. 2006;12(2):222-7.<https://doi.org/10.3748/wjg.v12.i2.222>
